# Toxicological Evaluation of Vanillin Flavor in E-Liquid Aerosols on Endothelial Cell Function: Findings from the Replica Project

**DOI:** 10.1007/s12012-026-10111-0

**Published:** 2026-04-13

**Authors:** R. Emma, A. Sun, K. Partsinevelos, S. Rust, V. Volarevic, R. Lesmana, A. Giordano, H. Goenawan, M. I. Barliana, A. Arsenijevic, N. Kastratovic, V. Markovic, B. Spasic, A. Distefano, L. Orlando, G. Carota, R. Polosa, Massimo Caruso, G. Li Volti

**Affiliations:** 1 Department of Clinical and Experimental Medicine, University of Catania, Via S. Sofia, 97, 95123 Catania, Italy; 2 Center of Excellence for the Acceleration of Harm Reduction (CoEHAR), University of Catania, Via S. Sofia, 97, 95123 Catania, Italy; 3 Present Address: Department of Medicine and Surgery, “Kore” University of Enna, 94100 Contrada Santa Panasia, Enna Italy; 4 Present Address: Department of Biology, College of Science and Technology, Temple University, Philadelphia, PA 19122 USA; 5 Present Address: Sbarro Institute for Cancer Research and Molecular Medicine, Center for Biotechnology, College of Science and Technology, Temple University, Philadelphia, PA 19122 USA; 6 Present Address: Department of Biomedical and Biotechnological Sciences, University of Catania, Via S. Sofia, 97, Catania, 95123 Italy; 7 ECLAT Srl, spin off of the University of Catania, Via. S Sofia 89, Catania, 95123 Italy; 8 Center for Harm Reduction of Biological and Chemical Hazards, Faculty of Medical Sciences, University of Kragujevac, 69 Svetozara Markovica 69, Kragujevac, 34000 Serbia; 9 Present Address: Department of Genetics, Faculty of Medical Sciences, University of Kragujevac, 69 Svetozar Markovic Street, Kragujevac, Serbia; 10 Department of Microbiology and Immunology, Faculty of Medical Sciences, University of Kragujevac, 69 Svetozar Markovic Street, Kragujevac, Serbia; 11 Present Address: Department of Biomedical Sciences, Faculty of Medicine, Universitas Padjadjaran, Bandung, Indonesia; 12 Division of Biological Activity, Central Laboratory, Universitas Padjadjaran, Bandung, Indonesia; 13 Center of Excellence for Pharmaceutical Care Innovation, Universitas Padjadjaran, Bandung, Indonesia; 14 Department of Medical Biotechnologies, University of Siena, Siena, Italy; 15 Present Address: Department of Biological Pharmacy, Faculty of Pharmacy, Universitas Padjadjaran, Jl. Ir. Soekarno Km 21, Jatinangor, 45363 Indonesia

**Keywords:** Flavor, Vanillin, E-liquids, Toxicity, Endothelial dysfunction, Cardiovascular effects

## Abstract

There are challenges that require collaboration among researchers to ensure that tobacco harm reduction strategies are evidence-based. A key challenge is evaluating the safety of flavors in e-cigarettes. Many flavorings are deemed “generally recognized as safe” (GRAS) for ingestion, but this does not guarantee their safety when inhaled. The Replica international research group is dedicated to the replication of studies on tobacco alternatives. As part of this effort, the group conducted a conceptual replication of the vanillin-specific experiments from the study by Fetterman et al. (2018), with the goal of validating their conclusions regarding this compound. We used Aspire Zelos 3 e-cigarette and prepared e-liquids containing propylene glycol, vegetable glycerin, and vanillin. The e-liquids were vaporized under regular (1 Ohm coil, 14 watts) and sub-Ohm (0.3 Ohm coil, 200 °C) settings using a vaping machine, following ISO20768:2018 puffing regime. The vapor was collected into a trapping solution to prepare aqueous extracts for treating human aortic endothelial cells. We evaluated cytotoxicity, oxidative stress, nitric oxide bioavailability, and inflammation addressing some gaps reported in the original study. We observed some harmful effects mostly attributable to ethanol used to trap vanillin, but no harmful effects from vanillin. Our results confirm the endothelial cell dysfunction observed in the original paper, but clarify that these effects are mainly attributable to ethanol and not to vaporized vanillin, indicating that, under the experimental conditions applied, vanillin does not elicit significant adverse effects on vascular endothelial cells. However, further research is needed to confirm its toxicological profile.

**Graphical Abstract:**

Created in BioRender. https://BioRender.com/rn8dt1m.

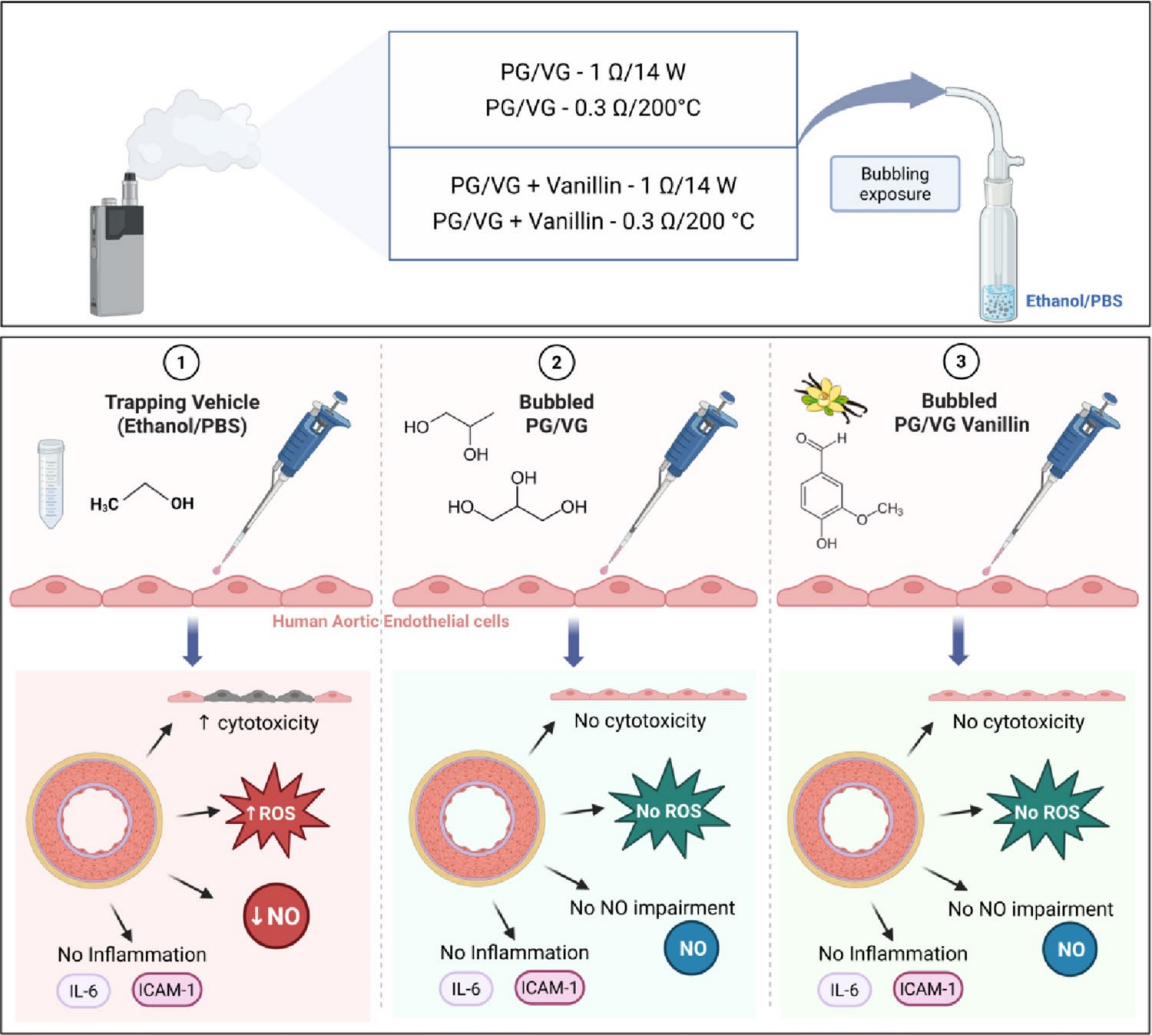

**Supplementary Information:**

The online version contains supplementary material available at 10.1007/s12012-026-10111-0.

## Introduction

Electronic cigarettes (ECs) have gained increased popularity among people who smoke for their potential for smoking cessation and harm reduction [1–3] due to their cost-effectiveness [4] and their ability to mimic the smoking experience without the production of harmful combustion or smoke [5, 6]. Research from five independent academic institutions, involved in a broader international initiative focused on the replicability of scientific studies, further supports the reduced harm associated with ECs relative to combusted cigarettes [7–9], even if the absolute risk of these products remains poorly explored and unclear. The consortium replicated studies examining the cytotoxic and inflammatory effects on human bronchial cells. Their findings concluded that aerosols from combustion-free nicotine delivery technologies are approximately 80% less cytotoxic compared to tobacco smoke [8]. Additionally, in a separate study, the consortium demonstrated that while tobacco combustion in cigarette smoke exhibited high levels of cytotoxicity, mutagenicity, and genotoxicity in vitro, the aerosol from ECs displayed modest or no such effects [9]. Finally, another study conducted by this consortium demonstrated a reduced impairment of repair mechanisms in vascular endothelium exposed to ECs and heated tobacco products (HTP) aerosol compared to combustion cigarette smoke [7]. Yet, despite the absence of tobacco combustion, the inhalation of these aerosols is not without potential risks.

E-cigarettes primarily consist of a battery and an atomizer where a liquid solution (commonly known as e-liquid or vape juice) is stored and vaporized by energy supplied to an electrical resistance. The liquid mainly contains propylene glycol (PG) and vegetable glycerin (VG), with or without nicotine. A significant characteristic of the e-cigarette market is the availability of a variety of flavorings in e-liquids. Besides tobacco-like flavors, consumers can choose flavors such as mint, fruits, desserts, candies beverages, and many more. It is estimated that several thousand e-liquid flavors have been identified [10, 11]. These flavors are fundamental components of the vaping experience and are created using a combination of food-grade flavors, PG, and VG. Client preferences for flavors can vary significantly, making the search for the right flavor a subjective and individual experience. In the largest survey ever performed on e-cigarette use, involving almost 70,000 participants, it was found that non-tobacco flavors, especially fruit and dessert flavors, significantly contribute to successful smoking cessation among adults who formerly smoked [12]. These flavors were also considered important not only in their effort to quit smoking but also in preventing relapse to cigarette smoking.

One of the most popular and concentrated flavoring chemicals in dessert-flavored e-liquids is vanillin. Vanillin has been approved as a food additive and is considered “generally recognized as safe” (GRAS) for certain uses in food. However, this does not in itself mean that the flavorings are safe when used via inhalation [13]. The food additive approval or GRAS status of a substance only applies to specific intended uses in the food and is not supported by studies that consider inhalation toxicity. In this regard, the toxicity of flavor chemicals should be re-evaluated, particularly regarding inhalation. Concerns have been raised about specific flavoring compounds, such as diacetyl, acetyl propionyl and acetoin, which were associated with respiratory problems if inhaled [13]. Many flavors used in e-liquids have been studied to determine their safety in vaping and tobacco products. Some studies suggest that certain flavors, including mixtures containing different forms of vanillin, may induce harmful effects when vaporized [14–18]. However, many of these studies do not accurately replicate the vaporization process that occurs in e-cigarettes.

To address this, we conducted a conceptual replication of the study by Fetterman et al. 2018 [16], employing standardized methods in accordance with ISO guidelines, ISO-compliant vaping machines, and commercially available e-cigarettes. Our goal was to assess whether the original findings, namely the ability of flavorings such as vanillin to compromise endothelial cell function, were reproducible under more realistic vaping conditions. As is customary in the Replica study, the experiments were independently performed in four international laboratories, following harmonized and standardized operating procedures (SOPs) [7–9].

## Methods

### Study Design and Harmonization Process

This is an interlaboratory in vitro study conducted in the framework of the new phase of Replica 2.0 project (https://replica.coehar.org/). The multi-center research network established for this study includes laboratories from Italy (LAB-A) – the leading center –, USA (LAB-B), Indonesia (LAB-C), and Serbia (LAB-D). This study is a conceptual replication of Fetterman et al. (2018). It maintains the fundamental scientific question of whether flavorings such as vanillin compromise endothelial cell function. At the same time, it addresses a second, equally relevant question: whether the methodological approach used in the original study is suitable for evaluating such effects under exposure conditions that mimic real-world vaping. We applied some improved methodological choices to enhance translational relevance and reproducibility. By replicating the biological question under more physiologically relevant exposure conditions and using standardized analytical protocols, this study contributes to verifying the robustness and generalizability of the original findings. We chose to replicate only the vanillin-specific arm of Fetterman et al.’s study, given the widespread use of vanillin in commercial e-liquids and its prominent toxicological profile in the original data. Vanillin was among the few flavoring agents that induced endothelial inflammation and nitric oxide impairment after aerosolization. These factors made vanillin a scientifically and clinically relevant candidate for focused replication. Moreover, replicating all flavoring conditions would have posed substantial logistical and technical challenges for a multicenter study.

As recommended by the Centre for Open Science transparency and openness promotion guidelines (https://www.cos.io/initiatives/top-guidelines), protocols were standardized across laboratories with SOPs defined for each experimental step. Moreover, all laboratories used the same cell line, cell-exposure equipment, and methods to assess endpoints. LAB-A arranged a kick-off meeting to train the staff of international partners and harmonize the SOPs. These SOPs have been designed to be as close as possible to the protocols of the original paper, except for the vanillin vaporization and cytotoxicity assay. Particularly, the major limitations stated by the authors of Fetterman et al. [16] were the use of flavor not diluted in propylene glycol (PG) and glycerol (VG), and their heating without using an electronic cigarette and a standardized exposure system. We covered these gaps by dissolving the vanillin in PG and VG solvents and using an electronic cigarette vaped by a standardized vaping machine (LM4E; Borgwaldt; Hamburg, Germany). Another difference from the original paper is the use of neutral red uptake (NRU) assay and MTS assay instead of TUNEL for the evaluation of cytotoxicity. While the TUNEL assay detects DNA fragmentation typically associated with apoptosis, it is not included in any OECD guidelines as a standard assay for evaluating cytotoxicity and is not considered suitable for regulatory toxicology purposes, as it detects a specific mechanism of cell death and may miss other forms such as necrosis or autophagy. We therefore adopted the NRU and MTS tests, which provide complementary and validated measures of cell viability and cytotoxicity, in order to align our protocol with standard toxicological frameworks and to better detect late-onset toxic effects after 24 h of exposure. In Table 1, we have summarized the critical steps of the methods used by Fetterman et al. and the corresponding method used by the Replica group.

**Table 1:** Comparison between Fetterman (2018) and Replica study methods

	Fetterman et al. [16]	Replica study
Vanillin test product	Vanillin diluted in ethanol as Sigma manufacturing instruction	Vanillin was first diluted with ethanol to a concentration of 2 M and then mixed with PG and VG
Flavor exposure	Drop-tube furnace set to temperatures 200 °C±50–700 °C±50 °C	Standardized vaping machine with e-cig containing PG/VG vanillin using two vaping setting (1.0 Ohm coil and 14 W; 0.3 Ohm coil and 200 °C)
Cytotoxicity Evaluation	Tunel assay after 90 min exposure	NRU and MTS assay after 24 h exposure
Oxidative Stress assessment	HAECs incubation (30 min) with DHE (10 μmol/L, Thermo Fisher) after 90 min flavor exposure	HAECs incubation (30 min) with DHE (10 μmol/L, Thermo Fisher) after 90 min flavor exposure
Nitric Oxide Bioavailability	HAECs incubation with 3 μmol/L DAF-2 DA (Calbiochem), after 90 min flavor exposure	HAECs incubation with 3 μmol/L DAF-2 DA (Sigma), after 90 min flavor exposure
Gene expression	HAECs exposed to flavors for 90 min + 90 min in media after flavoring exposure; RNA extraction using miRNeasy Micro Kit (Qiagen); cDNA reverse transcription kit (Quanta Bio, Beverly, MA); TaqMan Master Mix and TaqMan primers for IL-6 and ICAM-1 (Thermo Fisher).	HAECs exposed to flavors for 90 min + 90 min in media after flavoring exposure; RNA extraction using RNeasy Mini Kit (Qiagen); High-Capacity cDNA Reverse Transcription Kit (Thermo Fisher Scientific); TaqMan™ Fast Advanced Master Mix and TaqMan primers for IL-6 and ICAM-1 (Thermo Fisher).

### Cell Culture

The experiments were performed using the Human Aortic Endothelial Cells (HAEC) purchased from Lonza (CC-2535). Cells were cultured in EBM™−2 Basal Medium (Lonza, CC-3156) supplemented with EGM™−2 SingleQuots™ (Lonza, CC-4176), and incubated in a humidified atmosphere (5% CO_2_) at 37 °C. All experiments with HAECs were performed within passages 2 - 4. When the cells reached confluence, they were detached with 0.05% trypsin–0.02% EDTA solution and replated in new flasks (cell passaging), or into 96-well plates (cell viability assay, Nitric Oxide bioavailability, oxidative stress assessment), or into 12-well plates (RT-qPCR). The cells were received by the vendor in a frozen vial, and the thawed cells have been considered as “passage 0”.

### Test Products and Preparation of e-Liquids

The e-cigs used were Aspire Zelos 3 bought from Italian dealers. For the preparation of the e-liquids PG (Sigma Aldrich, P4347-500ML), VG (Sigma Aldrich, 1370282500), vanillin (Sigma Aldrich, V1104-100G), and absolute ethanol (Sigma Aldrich, 1009832500) were used. The day before the exposure run, four different e-liquids were prepared: PG/VG 50:50 (50% of PG and 50% of VG), PG/VG 50:50 with Vanillin (50% of PG and 50% of VG at 200 mM of vanillin), PG/VG 30:70 (30% of PG and 70% of VG), and PG/VG 30:70 with Vanillin (30% of PG and 70% of VG at 200 mM of vanillin). Vanillin was first diluted with absolute ethanol to a concentration of 2 M and then mixed with PG and VG. PG/VG 50:50 and PG/VG 50:50 with Vanillin were used with e-cig set in Wattage control mode (1.0 Ohm coil and 14 W) and the MTL (mouth-to-lung) drip tip inserted (Regular setting). PG/VG 30:70 and PG/VG 30:70 with Vanillin were used with e-cig set in Temperature control mode (0.3 Ohm coil and 200 °C) and the DTL (direct-to-lung) drip tip inserted (Sub-ohm setting).

### Vapor Generation and Aqueous Extract (AqEs) Preparation

E-cigarettes loaded with different e-liquids were vaped by the LM4E Vaping Machine (Borgwaldt, Hamburg, Germany) following the “CORESTA Reference Method n. 81” (CRM81) regimen (55 ml puff volume, drawn over 3 s, once every 30 s with square shaped profile, and with a puff velocity of 18.3 ml/s), accredited into ISO 20768:2018 [19]. A button pre-activation of 1 s was also applied as per guidelines CRM81. The standard exhaust time for LM4E was 0.7 s with a flow rate in the impinger of 78.57 ml/s. For each e-liquid and experimental setting, 100 puffs were generated using a single device and divided into two consecutive runs of 50 puffs to prevent dry-puff generation. The resulting vapor was bubbled into an impinger containing 30 ml of trapping solution (20% absolute ethanol and 80% PBS) to prepare the AqEs for subsequent treatments. The laboratory conditions were checked using temperature and humidity sensors prior and during the vapor generation, maintaining a relative humidity of 40–70% and a temperature between 15 °C and 25 °C ± 2 °C, according to ISO 20,768 [19]. It is important to note that the vehicle solution (20% ethanol in PBS), used solely as the trapping medium for aerosol collection, was not vaporized and thus remained chemically unaffected by the e-cigarette settings. Consequently, any influence of vaporization parameters (e.g., sub-ohm vs. regular) on the composition of the vehicle itself is excluded. To account for the potential biological effects of ethanol, matched vehicle controls at the same concentrations found in the AqEs were included in all experiments to isolate any contribution of the trapping medium to the observed outcomes.

### Neutral Red Uptake (NRU) Assay

24 h prior to AqEs treatment, HAECs were trypsinized (0.05% Trypsin/0.02% EDTA), resuspended in EGM™−2 complete medium, seeded in a 96-well plate at a density of 10 × 10^3^ viable cells/well, and incubated at 37 °C (5% CO2, humidified atmosphere). After vapor generation, the obtained AqEs were filtered with 0.2 μm syringe filter. Then the cells were treated for 24 h with seven different dilutions of PG/VG 50:50 AqE, PG/VG 50:50 with Vanillin AqE, PG/VG 30:70 AqE, and PG/VG 30:70 with Vanillin AqE (1:2 or 100 mM, 1:4 or 50 mM, 1:8 or 25 mM, 1:20 or 10 mM, 1:200 or 1 mM, 1:2000 or 0.1 mM, 1:20000 or 0.01 mM). Following Fetterman’s indications [16], the molarity concentration reported and the relative dilutions of the AqE are referred to the initial concentration of Vanillin (200 mM) calculated in the relative liquids. For the dilution of AqEs EGM™−2 complete medium with 20 mM HEPES (Gibco, 15630-080) was used. Moreover, the experiments included a negative control consisting of cells with culture media (EGM™−2 complete medium with 20 mM HEPES), seven vehicle controls with cells treated with Ethanol/PBS at the same concentrations as AqE treatments (1:2 or 100 mM, 1:4 or 50 mM, 1:8 or 25 mM, 1:20 or 10 mM, 1:200 or 1 mM, 1:2000 or 0.1 mM, 1:20000 or 0.01 mM), and a positive control with 350 μM sodium dodecyl sulfate (SDS). After the treatments, the cells were washed with PBS and incubated with Neutral Red Solution (0.05 g/L; Sigma, N2889-100ML) diluted in EGM™−2 complete medium with 20 mM HEPES at 37 °C (5% CO2, humidified atmosphere) for 3 hours. Afterwards, the cells were washed with PBS, and the Neutral Red dye retained by cells was extracted by the addition of destaining solution (ethanol, distilled water, and acetic acid at a ratio of 50:49:1, respectively). Subsequently, the plates were shaken using an orbital agitator for 10 min at 300 rpm. Eventually, Neutral Red absorbance was measured in a microplate reader at 540 nm. NRU data were normalized to control medium or to vehicle control.

### MTS Assay

Cell seeding and treatment procedures were performed as previously stated for the NRU assay. After 24 h of incubation with AqEs and controls (negative control, vehicle controls, and positive control), the treatment was removed from the wells and the cells were incubated with 100 μl of EGM™−2 complete medium with 20 mM HEPES and 20 μl of MTS (Promega, CellTiter 96^®^ AQueous One Solution Reagent) at 37 °C (5% CO2, humidified atmosphere) for 3 h. The absorbance of soluble formazan produced by cellular reduction of MTS was measured using a microplate reader at 490 nm. MTS data were normalized to control medium or to vehicle control.

### Oxidative Stress Assessment

HAECs were seeded in a 96-well plate at a density of 10 × 10^3^ cells/well and incubated at 37 °C (5% CO2, humidified atmosphere) for 24 h, until 80–90% confluence. Then the cells were exposed for 90 min to four different dilutions (1:20 or 10 mM, 1:200 or 1 mM, 1:2000 or 0.1 mM, and 1:20000 or 0.01 mM) of PG/VG 50:50 AqE, PG/VG 50:50 with Vanillin AqE, PG/VG 30:70 AqE, PG/VG 30:70 with Vanillin AqE, and the corresponding vehicle controls (Ethanol/PBS). The reported molarity concentration and dilutions of AqEs were established basing on the initial Vanillin concentration of the related products (200 mM). Prior to dilution, AqEs were filtered with 0.2 μm syringe filter. For the dilution, EGM™−2 complete medium with 20 mM HEPES was used. Next, HAECs were incubated for 30 min with 10 μmol/L dihydroethidium (DHE; Thermo Fisher Scientific, D11347). After that, HAECs were washed 3 times with pre-warmed DPBS to remove DHE. A negative control (EGM™−2 complete medium with 20 mM HEPES) and a positive control consisting of 50 μmol/L Antimycin A (Sigma-Aldrich, A8674) were included. The fluorescence intensity was measured within 30 min using a microplate reader with excitation of 518 nm and emission of 606 nm. Data are shown as fold change in DHE fluorescence in comparison with vehicle control.

### Nitric Oxide Bioavailability

Cell seeding and exposure to AqE solutions were performed as explained above (Oxidative Stress assessment section). Next, the cells were incubated for 30 min with 3 µmol/L 4,5-diaminofluorescein diacetate (DAF-2 DA; Sigma-Aldrich, D225). After that, the cells were washed 2 times with pre-warmed DPBS, stimulated for 15 min with 1 µmol/L Calcium Ionophore A23187 (Sigma-Aldrich, C7522), and fixed with 2% paraformaldehyde in DPBS for 10 min at room temperature. Afterwards, paraformaldehyde was replaced with 200 μl of DPBS to each well and the fluorescence intensity (excitation of 492 nm, emission peak at 515 nm) was measured by a microplate reader. Nitric Oxide data were presented as percentage increase in DAF-2 DA fluorescence stimulated by Calcium Ionophore A23187 compared with unstimulated cells.

### Gene Expression

HAECs were seeded in 12-well plates at a density of 100 × 10^3^ cells/well and incubated at 37 °C (5% CO2, humidified atmosphere) for 24 h, until 80–90% confluence. Then the cells were exposed to 1:20, 1:200, 1:2000, and 1:20000 dilutions of AqEs. AqE solutions were prepared as previously described for oxidative stress assessment. Next, the cells were incubated for 90 min with complete medium (20 mM HEPES) to allow changes in RNA expression. For cell disruption and RNA isolation the RNeasy Mini Kit (Qiagen) was used, following the instructions provided by the manufacturer. RNA purity and quantification were performed using spectrophotometric absorbance measurements at 260 nm and 280 nm. cDNA synthesis of the isolated RNA was carried out with the High-Capacity cDNA Reverse Transcription Kit (Thermo Fisher Scientific) according to the manufacturer’s protocol. Quantitative Real-Time PCR was performed using the TaqMan™ Fast Advanced Master Mix (Thermo Fisher Scientific) following the manufacturer’s instruction manual. Glyceraldehyde 3-phosphate dehydrogenase (GAPDH) was used as the reference gene, in accordance with the original study replicated here, to ensure methodological consistency and comparability of the results. GAPDH, IL-6, and ICAM-1 TaqMan™ probes were purchased from Thermo Fisher Scientific. The 2^−∆∆Ct^ was calculated from cycle threshold (Ct) values, after normalization to GAPDH as housekeeping gene.

### Statistics

Microsoft Excel was used to tabulate and process all of the raw data. The Shapiro-Wilk test was used to assess the normality or skewness of data distribution. Correlation analyses were performed to evaluate the relationship between the results of each laboratory. Pearson’s correlation analysis was conducted for symmetrical data, while Spearman’s Rank correlation analysis was used for skewed data. Moreover, the intra-class correlation coefficient (ICC) was computed using an absolute-agreement, two-way mixed-effects model to evaluate the agreement in the repeatability of the intrasession measurements among the laboratory results. R version 4.2.3 (2023-03-15) was utilized for reproducibility analyses, including the generation of correlation plots. The outlier detection was made using the robust regression-based outlier rejection (ROUT) test. All data were reported as median (Interquartile range – IQR). The Kruskal-Wallis test was applied to determine any statistically significant differences between the medians of study groups. Additionally, post-hoc multiple comparisons were conducted using Dunn’s test or pairwise Wilcoxon rank-sum test with Bonferroni correction to examine group differences further. All analyses were considered significant with a p-value of less than 5%. GraphPad Prism 8 software was used for data analysis and the generation of graphs unless otherwise stated.

## Results

### Interlaboratory Reproducibility

The results of the Intraclass Correlation Coefficient (ICC) calculations using an absolute-agreement, two-way mixed-effects model are presented in Table 2.

**Table 2:** Results of ICC calculation using absolute-agreement, two-way mixed-effects model

			95% Confidence interval		F-Test with true value 0			
	Raters (LAB)	ICC	Lower bound	Upper bound	Value	Df1	Df2	*p* value
NRU (regular)	4 (A; B;C; D)	0.167	0.009	0.4	3.62	21	10.9	0.016
NRU (subohm)	4 (A; B;C; D)	0.251	0.028	0.525	6.29	21	7.26	0.008
MTS (regular)	2 (A; B)	0.341	−0.109	0.694	3.99	21	3.8	0.101
MTS (subohm)	2 (A; B)	0.949	0.736	0.984	63.9	21	5.1	< 0.0001
OxS (regular)	4 (A; B;C; D)	0.074	−0.018	0.279	2.1	13	17.1	0.076
OxS (subohm)	4 (A; B;C; D)	0.052	−0.037	0.256	1.53	13	32.1	0.159
NO (regular)	4 (A; B;C; D)	−0.08	−0.132	0.092	0.375	11	3.75	0.893
NO (subohm)	4 (A; B;C; D)	0.017	−0.038	0.175	1.23	11	34.7	0.303
IL-6 (regular)	4 (A; B;C; D)	0.014	−0.059	0.2	1.13	12	39	0.367
IL-6 (subohm)	4 (A; B;C; D)	0.108	−0.021	0.371	2.12	12	21.5	0.063
ICAM-1 (regular)	4 (A; B;C; D)	−0.007	−0.036	0.078	0.865	12	24.7	0.590
ICAM-1 (subohm)	4 (A; B;C; D)	−0.0003	−0.054	0.147	0.997	12	35.9	0.471

The table includes the ICC values, 95% confidence intervals, F-test values, degrees of freedom (Df1 and Df2), and p-values for each group of raters

The correlations of NRU results among all laboratories are shown in Figure S1 of supplementary information. Significant correlations were observed among all laboratories for the regular setting, except for the correlation between LAB-A and LAB-D. Strong correlations were also observed for the sub-ohm setting across all laboratories. Also, the NRU for regular setting had an ICC of 0.167, which was statistically significant (*p*= 0.016). Similarly, the NRU (sub-Ohm) group had an ICC of 0.251, also significant (*p*= 0.008).

For the MTS (regular) group, while correlation indicates that there is a good relationship (rho= 0.483, *p*= 0.024) between the measurements of the two centers (Fig. S2A), the nonsignificant ICC of 0.341 (*p*= 0.101) indicates that this relationship is not strong enough to ensure good reproducibility. In contrast, the MTS (sub-Ohm) group showed high correlation coefficient (rho= 0.896) (Fig. S2B) and high ICC of 0.949, indicating highly significant results (*p*< 0.0001).

Poor correlation was observed for Oxidative stress, Nitric oxide, IL-6 and ICAM-1 assessments for both regular and sub-ohm setting, as showed respectively in Figure S3, S4, S5 and S6 of supplementary information. Also, the ICC values indicated poor agreement among laboratories when performing oxidative stress and nitric oxide evaluations. Indeed, the oxidative stress (regular) group showed an ICC of 0.074, which was not significant (*p*= 0.076). The oxidative stress (sub-Ohm) group had an ICC of 0.052, also not significant (*p*= 0.159). For the NO (regular) group, the ICC was −0.08, which was not significant (*p*= 0.893). The NO (sub-Ohm) group had a low ICC of 0.017, which was also not significant (*p*= 0.303). For the IL-6 (regular) group, the ICC was 0.014, which was not statistically significant (*p* = 0.367). The IL-6 (sub-Ohm) group had a slightly higher ICC of 0.108, which approached significance (*p* = 0.063). For the ICAM-1 (regular) group, the ICC was −0.007, which was not significant (*p* = 0.590). Similarly, the ICAM-1 (sub-Ohm) group had a very low ICC of −0.0003, also not significant (*p* = 0.471).

These results indicate that the reliability of the measurements varied across the different evaluations and settings, with the NRU (both regular and sub-ohm) and MTS (sub-ohm) assays showing the highest reliability.

### Cytotoxicity Evaluation of Vanillin

**Fig. 1:**
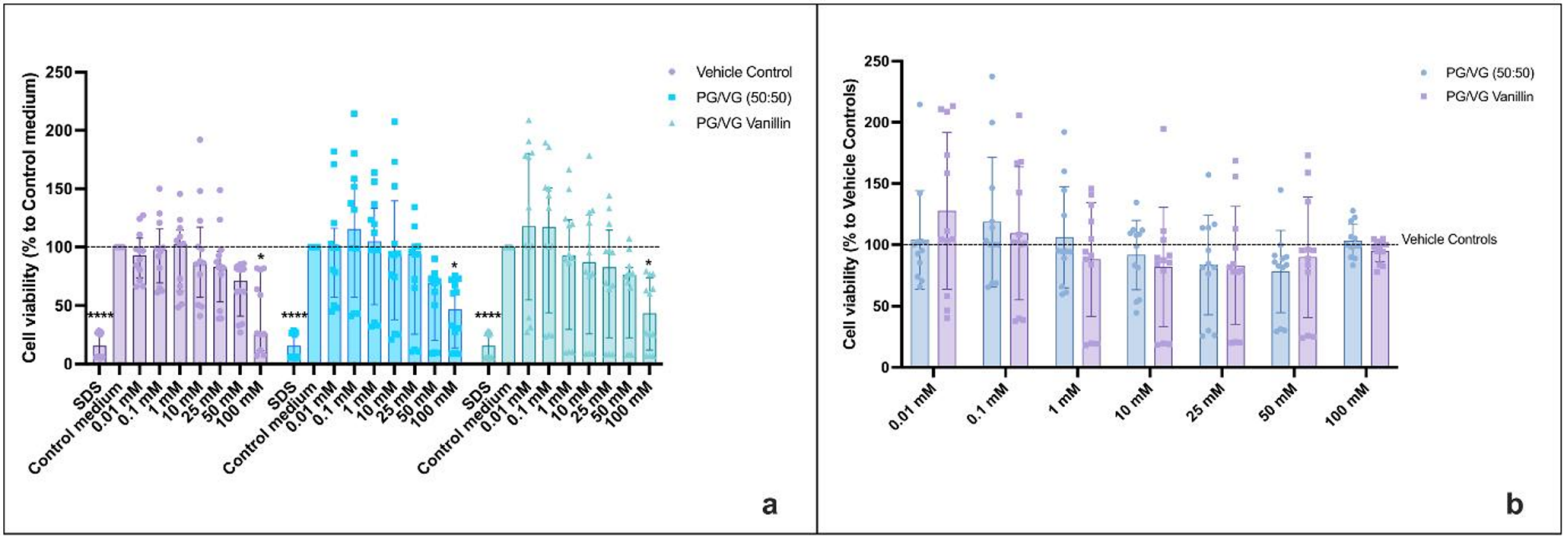
HAECs cytotoxicity evaluation by NRU assay of PG/VG base and PG/VG Vanillin using the regular setting at 24 h. **a** NRU data normalized as percentage of control medium. The dashed line corresponds to control medium. P values are reported relative to the control medium. **b** NRU data normalized as percentage of corresponding Vehicle control concentration. The dashed line corresponds to Vehicle control. All data are reported as median (IQR). * *p*< 0.05; ****p< 0.0001

**Fig. 2:**
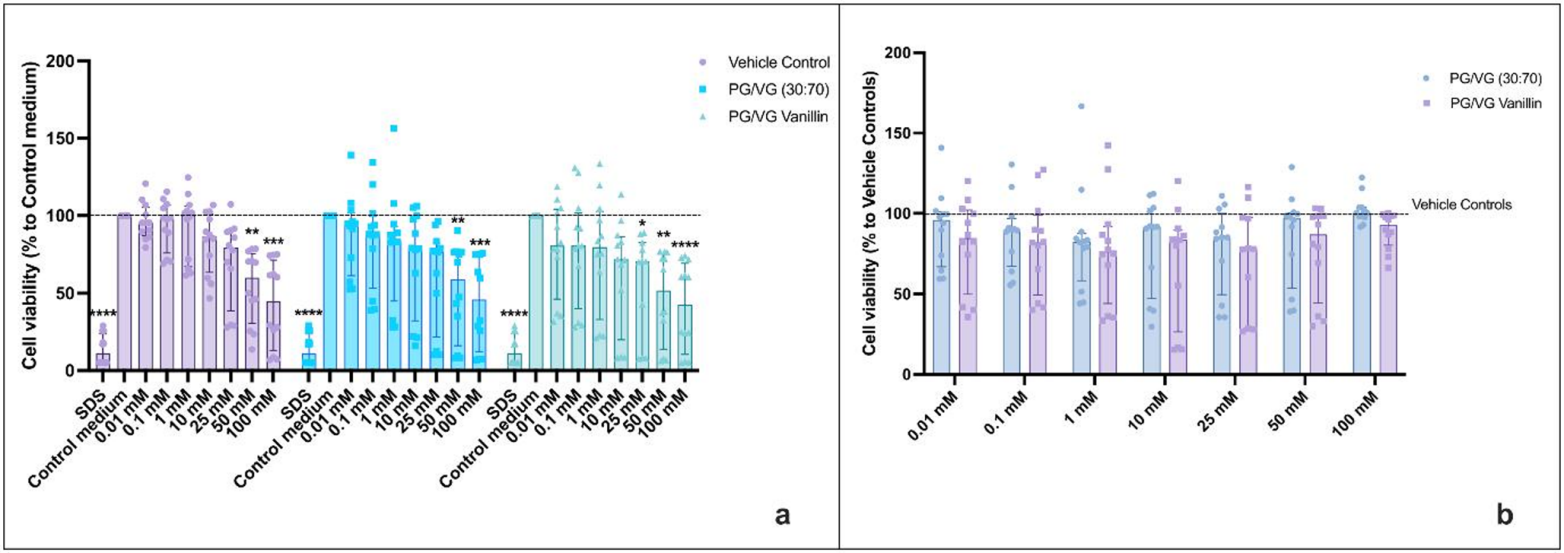
HAECs cytotoxicity evaluation by NRU assay of PG/VG base and PG/VG Vanillin using the sub-ohm setting at 24 h. **a** NRU data normalized as percentage of control medium. The dashed line corresponds to control medium. P values are reported relative to the control medium. **b** NRU data normalized as percentage of corresponding Vehicle control concentration. The dashed line corresponds to Vehicle control. All data are reported as median (IQR). *p< 0.05; ** *p*< 0.01; ***p< 0.001; ****p< 0.0001

Cytotoxicity of HAECs exposed to PG/VG Vanillin for both regular and sub-ohm settings was evaluated by NRU and MTS assays after 24 h of treatment, in contrast to Fetterman’s assessment at 90 min. The evaluation of cytotoxicity by NRU showed a significant decrease in HAECs viability induced by the positive control (SDS 350 μM) (*p*< 0.0001) (Figs. 1a and 2a). Using regular setting, we observed significant cell viability reduction after exposure to PG/VG base (*p*= 0.028) and PG/VG Vanillin (*p*= 0.02) compared to control medium. Also, 100 mM of Vehicle control showed significant decrease in cell viability compared to control medium (*p*= 0.038). No significant differences were observed between each concentration of PG/VG base and PG/VG Vanillin, and with the corresponding vehicle control concentrations (Fig. 1a). NRU data normalized to vehicle control (Fig 1b) revealed no significant differences in cell viability among PG/VG base and PG/VG Vanillin compared at each concentration (Fig 1b) and compared to vehicle controls.

Under sub-ohm conditions, the AqEs generated from PG/VG base and PG/VG Vanillin caused significant decreases in HAEC viability at 100 mM (*p* = 0.0006 and *p* < 0.0001, respectively) and 50 mM (*p* = 0.008 and *p* < 0.002, respectively), and for PG/VG Vanillin also at 25 mM (*p* = 0.042), compared to the control medium. Similar reductions were observed for the corresponding vehicle control solutions, which were not vaporized but prepared at ethanol concentrations equivalent to those found in the AqEs (Fig. 2a). As with the regular setting, no significant differences were observed between each concentration of PG/VG base and PG/VG vanillin, as well as the corresponding vehicle control concentrations in the sub-ohm setting (Fig. 2a). The NRU data normalization to vehicle control (Fig. 2b) indicated no significant differences in cell viability between the corresponding PG/VG base and PG/VG Vanillin concentrations and versus the vehicle controls (Fig 2b).

**Fig. 3:**
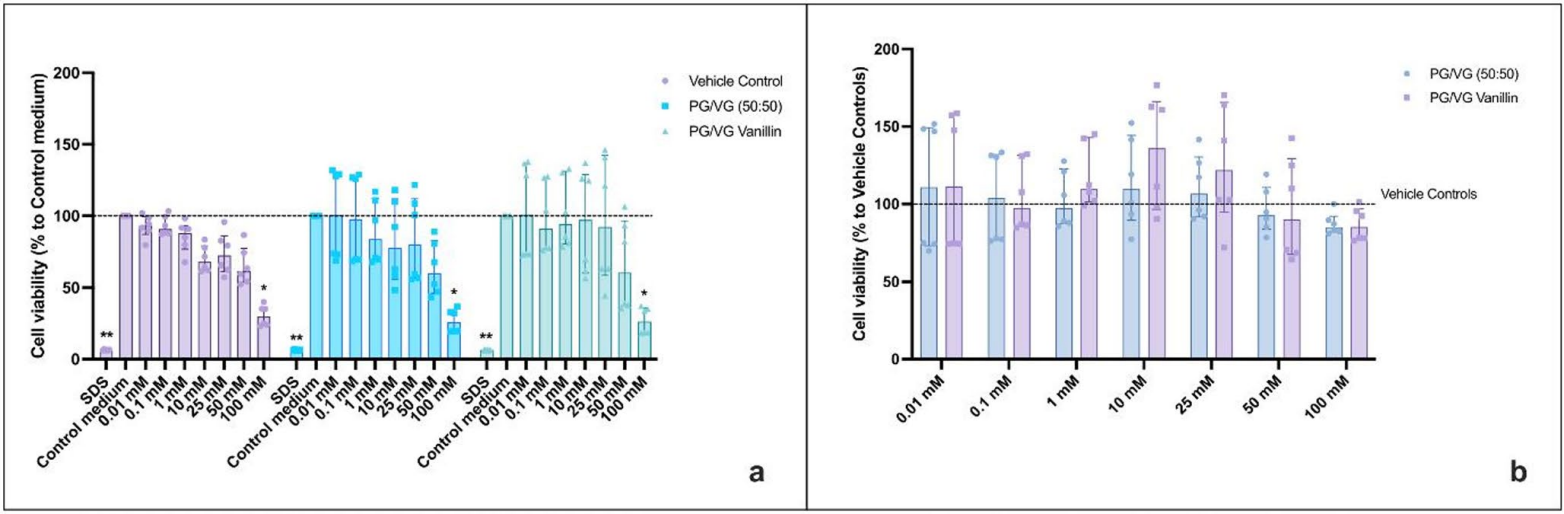
HAECs cytotoxicity evaluation by MTS assay of PG/VG base and PG/VG Vanillin using the regular setting at 24 h. **a** MTS data normalized as percentage of control medium. The dashed line corresponds to control medium. P values are reported relative to the control medium. **b** MTS data normalized as percentage of corresponding Vehicle control concentration. The dashed line corresponds to Vehicle control. All data are reported as median (IQR). *p< 0.05; ** *p*< 0.01

**Fig. 4:**
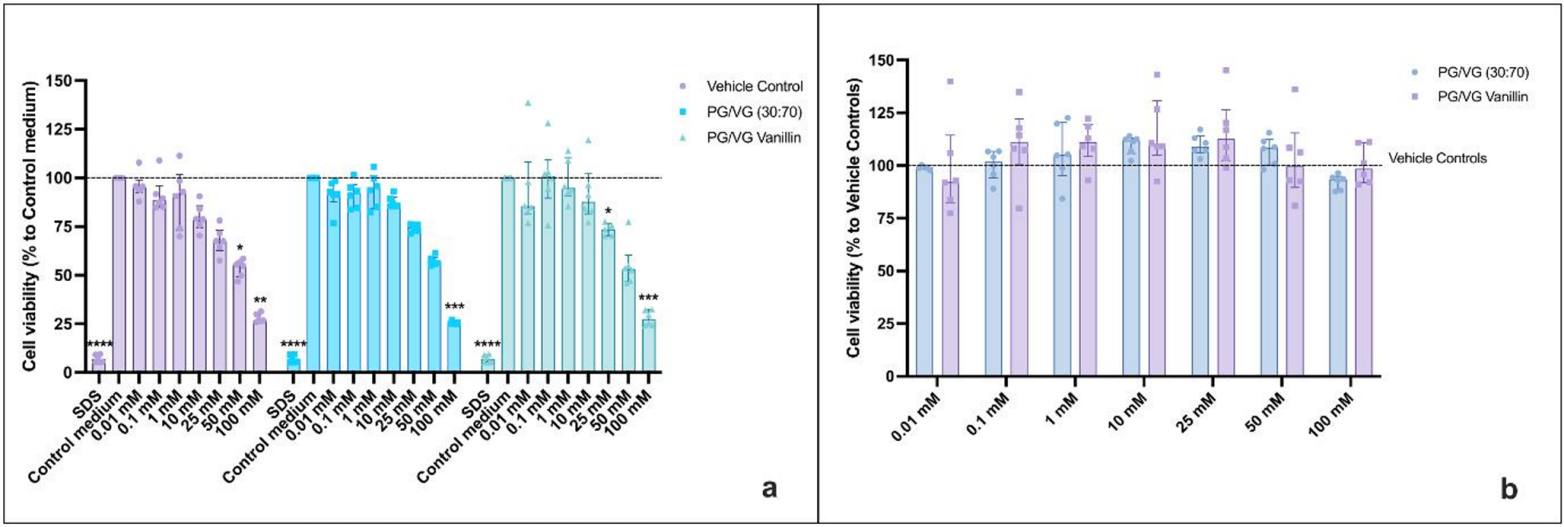
HAECs cytotoxicity evaluation by MTS assay of PG/VG base and PG/VG Vanillin using the sub-ohm setting at 24 h. **a** MTS data normalized as percentage of control medium. The dashed line corresponds to control medium. P values are reported relative to the control medium. **b** MTS data normalized as percentage of corresponding Vehicle control concentration. The dashed line corresponds to Vehicle control. All data are reported as median (IQR). *p< 0.05; ** *p*< 0.01; ***p< 0.001; ****p< 0.0001

Similar results were observed when the MTS assay was used for the cytotoxicity evaluation in HAECs exposed to PG/VG and PG/VG Vanillin. The MTS assay was carried out only by LAB-A and LAB-B, thus having two independent replicants of each experiment. The positive control (SDS 350 μM) caused a significant reduction in HAEC viability (*p* = 0.0031 and *p* < 0.0001, respectively; Figs. 3a and 4a), confirming the responsiveness of the assay. Under regular conditions, the AqEs generated from PG/VG base and PG/VG Vanillin caused a reduction in HAEC viability at the 100 mM concentration (*p* = 0.0259 and *p* = 0.0234, respectively) compared to the control medium. A similar decrease was observed for the corresponding vehicle control solution (*p* = 0.0472). No significant differences were found between the AqEs and their respective vehicle controls at any concentration (Fig. 3a). Normalizing the MTS data to the vehicle control (Fig. 3b) revealed no significant differences among the test products and their corresponding vehicle controls. Additionally, no notable differences in cell viability were observed when comparing PV/VG base and PG/VG Vanillin at each concentration (Fig. 3b).

Exposure to AqEs produced under sub-ohm conditions also resulted in reduction of HAEC viability compared to the regular setting. Significant decreases were detected at 100 mM for PG/VG base and PG/VG Vanillin (*p* = 0.0004 and *p* = 0.0008, respectively), and at 50 mM for PG/VG Vanillin (*p* < 0.0316). Comparable effects were also observed with the vehicle control solutions (*p* = 0.0013 and *p* = 0.0234 at 100 and 50 mM, respectively). Although the 50 mM PG/VG base exhibited a similar trend to the vehicle control and PG/VG Vanillin, the difference did not reach statistical significance (*p* = 0.051). No significant differences were observed between the PG/VG base and PG/VG Vanillin AqEs or their respective vehicle controls (Fig. 4a). Normalizing the MTS data to the vehicle control (Fig. 4b) revealed no significant differences among the test products and their respective vehicle controls. Furthermore, the comparison between PG/VG base and PG/VG Vanillin at each concentration indicated no significant variations in cell viability (Fig. 4b).

All these results demonstrate that the cytotoxic effect on HAECs is predominantly due to the ethanol present in the vehicle control. In fact, normalization of the data to the respective ethanol concentrations reveals no cytotoxic effect of the aerosols with and without vanillin.

### Vanillin Did Not Induce Oxidative Stress

**Fig. 5:**
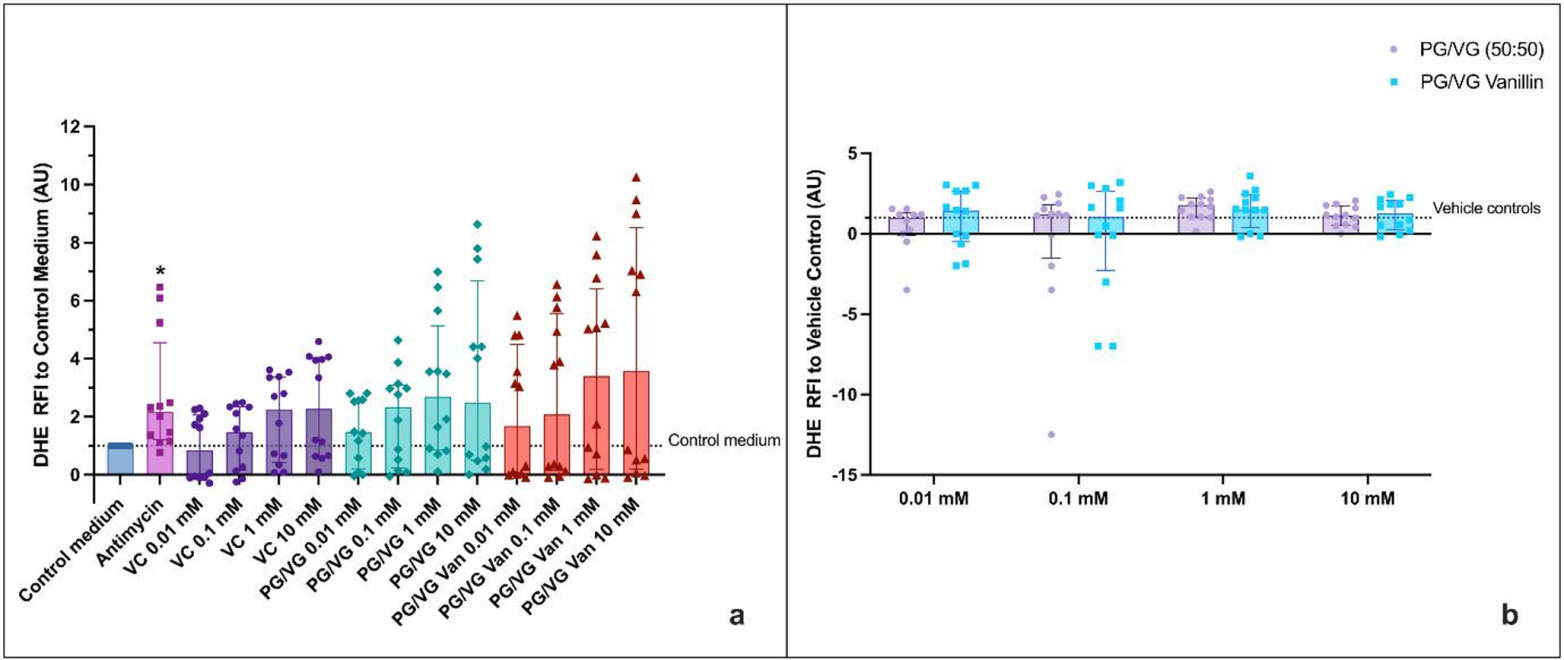
HAECs Oxidative Stress (OxS) evaluation by DHE of PG/VG base and PG/VG Vanillin using the regular setting. **a** OxS data normalized to control medium. The dashed line corresponds to control medium. P values are reported relative to the control medium. **b** OxS data normalized to the corresponding Vehicle control concentration. The dashed line corresponds to Vehicle control. All data are reported as median (IQR). VC: Vehicle control; *p< 0.05

**Fig. 6:**
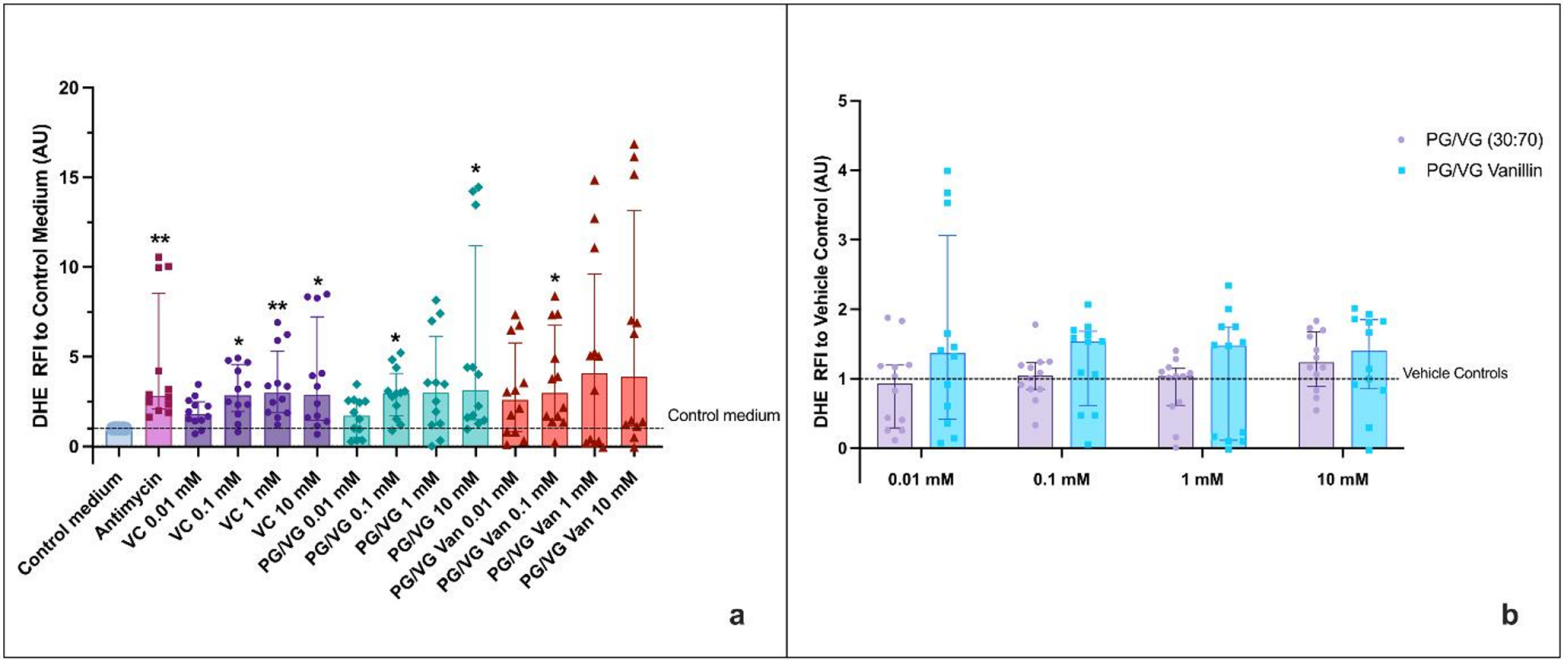
HAECs Oxidative Stress (OxS) evaluation by DHE of PG/VG base and PG/VG Vanillin using the sub-ohm setting. **a** OxS data normalized to control medium. The dashed line corresponds to control medium. P values are reported relative to the control medium. **b** OxS data normalized to the corresponding Vehicle control concentration. The dashed line corresponds to Vehicle control. All data are reported as median (IQR). VC: Vehicle control; *p< 0.05, **p< 0.01

Following 90 min HAECs’ treatment with PG/VG and PG/VG Vanillin at various doses, oxidative damage was measured using the fluorescent dye DHE, as reported by Fetterman and colleagues [16]. The results confirm the efficacy of antimycin in inducing oxidative stress in both experimental settings. Since ethanol has been reported to increase reactive oxygen species (ROS) [20], evaluation of Vehicle Controls per se was performed. As a result, an increase in oxidative stress levels was observed in cells exposed to the corresponding vehicle control solutions (non-vaporized ethanol/PBS at matched concentrations) (Fig. S8 and S9). The assessment of oxidative stress for the regular setting revealed no significant difference between the different conditions tested in comparison to the medium control (Fig. 5a). Although a slight increase in oxidative stress was observed in cells exposed to PG/VG Vanillin AqEs compared to control medium, the high variability of the data likely prevented statistical significance. In contrast, exposure to AqEs generated under sub-ohm conditions resulted in a statistically significant increase in oxidative stress for PG/VG at 0.1 mM and 10 mM (*p*< 0.05) and PG/VG vanillin at 0.1 mM (*p*< 0.05) compared to control medium (Fig. 6a). As previously mentioned, the data are characterized by wide interquartile ranges, indicating a high degree of variability within each group. Moreover, the data normalization to the corresponding Vehicle control concentrations (Figs. 5b and 6b) indicated no significant differences between unflavored PG/VG and PG/VG Vanillin compared to the respective Vehicle control concentration. In addition, no significant variations were detected between PG/VG and PG/VG Vanillin when evaluated at each concentration level.

### Nitric Oxide Bioavailability was not Impaired by Vanillin

**Fig. 7:**
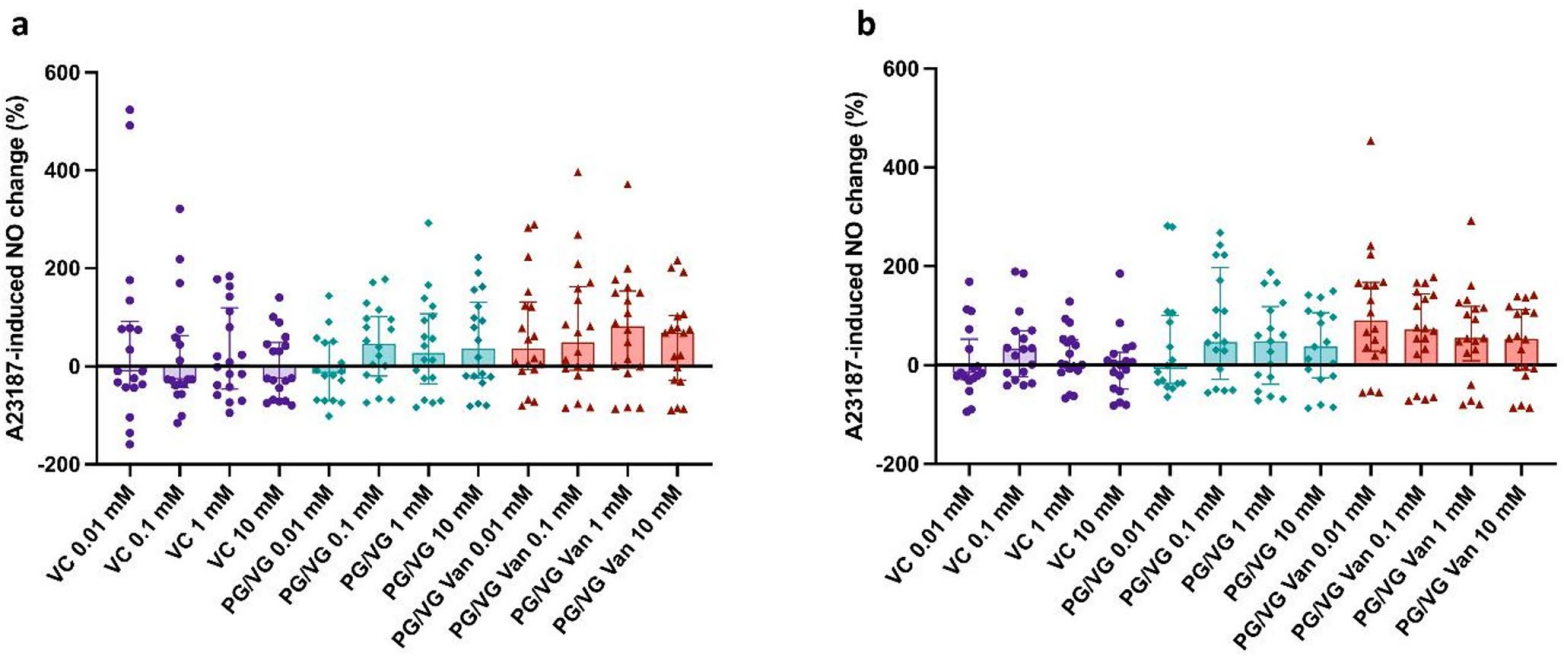
Effects of PG/VG and PG/VG Vanillin on A23187-stimulated nitric oxide production in human aortic endothelial cells (HAECs) using Regular Setting (**a**) and sub-Ohm setting (***b***). All data are normalized as percentage of A23187-induced NO change and reported as median (IQR). VC: Vehicle control

As carried out by Fetterman and colleagues [16], we evaluated the effect of vanillin on nitric oxide production by HAECs in response to A23187 stimulation. No significant differences were observed between PG/VG and PG/VG Vanillin and the respective Vehicle Control concentrations for both regular (Fig. 7a) and sub-ohm settings (Fig. 7b). In some cases, it appears that only PG/VG exposure may exert a minor impact on NO production, though this is not significant compared to vehicle control. Instead, the exposure to PG/VG Vanillin appears to improve the bioavailability of NO compared to PG/VG, although no significant differences were observed among PG/VG and PG/VG Vanillin.

### Gene Expression of IL-6 and ICAM-1

**Fig. 8:**
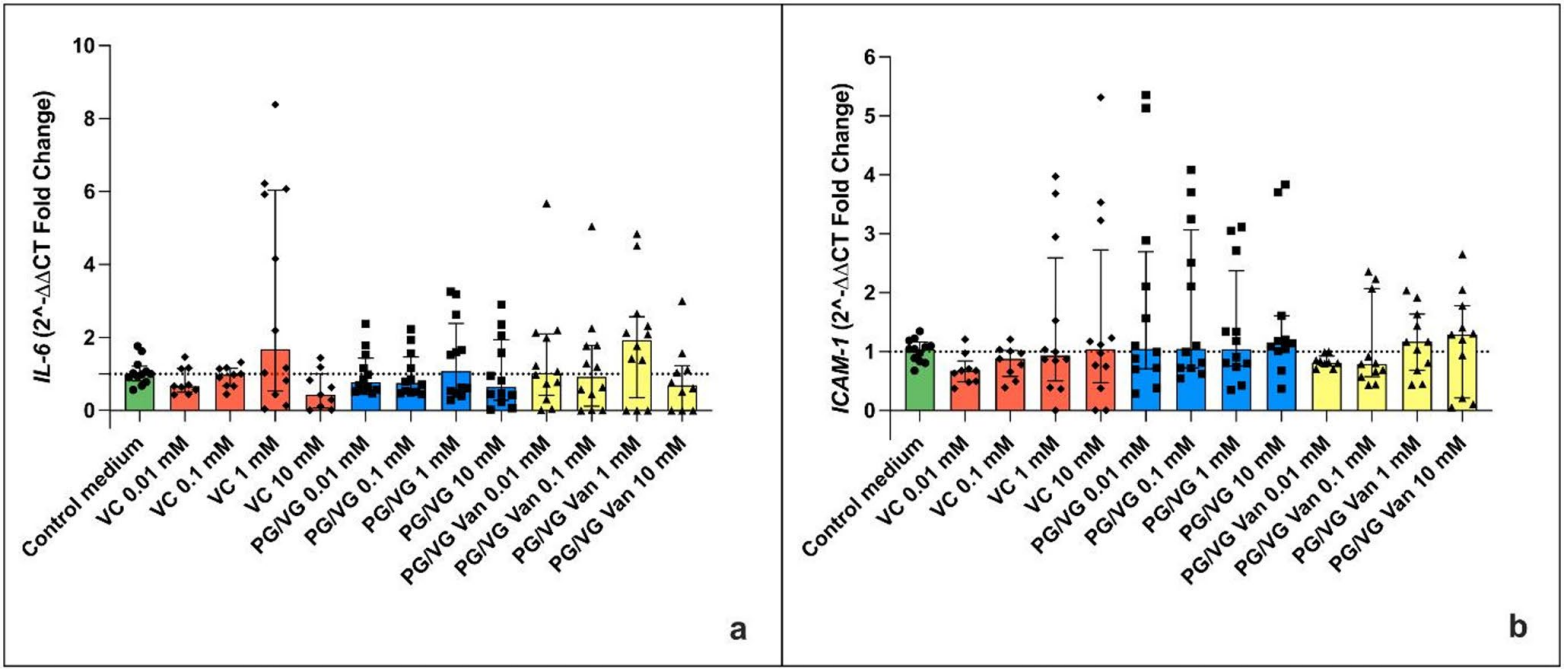
Gene expression of IL-6 (**a**) and ICAM-1 (**b**) in aortic endothelial cells under different treatments produced using the Regular setting. All data are reported as median (IQR). VC: Vehicle control

**Fig. 9:**
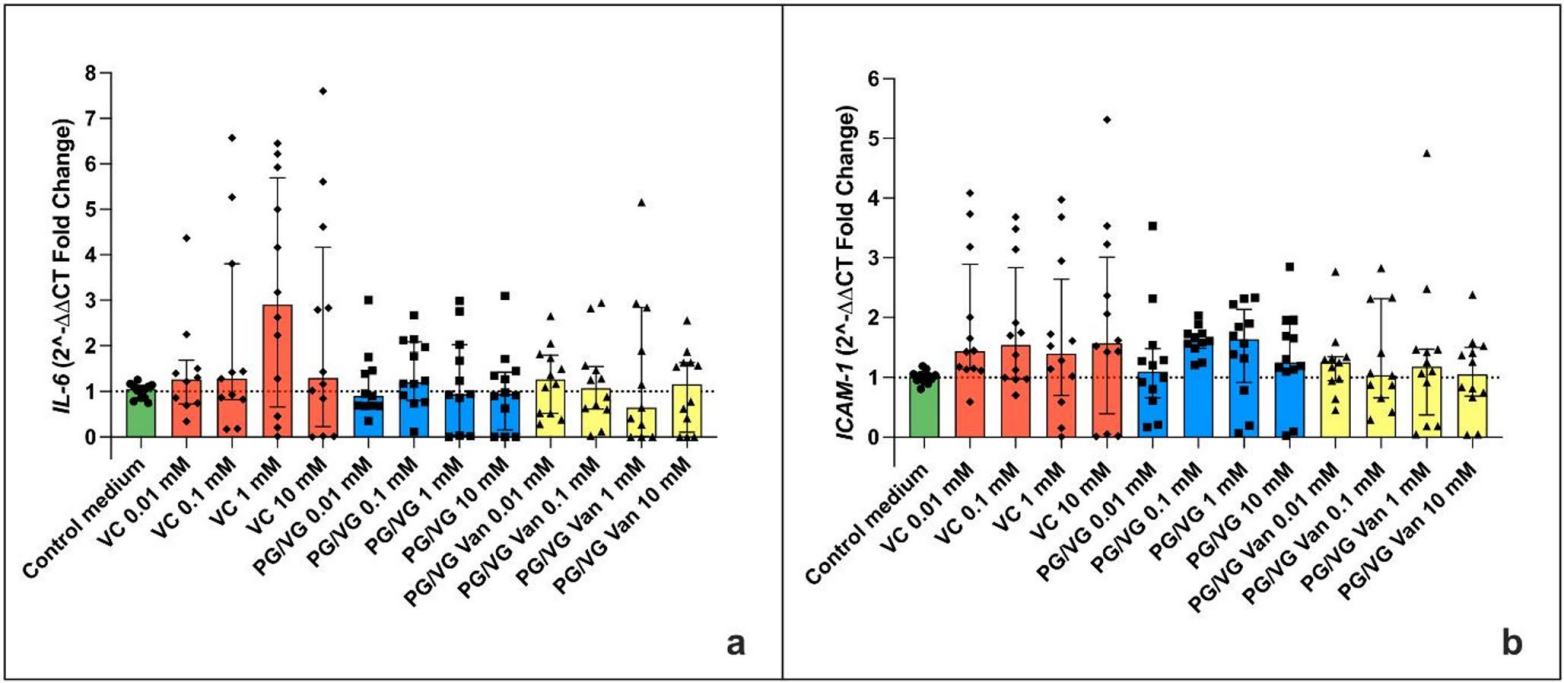
Gene expression of IL-6 (**a**) and ICAM-1 **(b**) in aortic endothelial cells under different treatments produced using the Subohm setting. All data are reported as median (IQR). VC: Vehicle control

The evaluation of IL-6 gene expression in aortic endothelial cells treated under the experimental conditions showed no statistically significant variations compared to the control. Both PG/VG and PG/VG Vanillin treatments did not show significant differences in IL-6 expression compared to the control. This result was observed for both the Regular (Fig. 8a) and Sub-ohm (Fig. 9a) experimental setups. Similarly, the gene expression of ICAM-1 did not show statistically significant variations in aortic endothelial cells treated with Vehicle Control, PG/VG, and PG/VG Vanillin at the various concentrations (10 mM, 1 mM, 0.1 mM, and 0.01 mM). Again, no significant differences in ICAM-1 expression were observed compared to the control in both the Regular (Fig. 8b) and Sub-ohm (Fig. 9b) setups.

## Discussion

The aim of the present study was to conceptually replicate the findings by Fetterman et al., 2018 [16], with a specific focus on evaluating the impact of vanillin, an aromatic compound commonly used in e-liquids, on endothelial cell function. We evaluated the same endpoints as in Fetterman’s study, including cytotoxicity, oxidative stress, and nitric oxide bioavailability. While the original study provided evidence that vanillin flavoring induces some acute alterations in endothelial functions, our replication efforts revealed different results and covered some methodological gaps mainly concerning the method of exposure.

A key aspect of our replication was the careful alignment of our methodology with that described in the original study by Fetterman et al., as well as with additional details provided directly by the corresponding author. Despite these efforts, we identified some methodological differences that could partly explain the discrepancies in our findings. Notably, while the original study used the TUNEL assay to assess cytotoxicity 90 min after exposure, this method primarily detects DNA fragmentation associated with apoptosis, representing only one form of cell death [21]. Notwithstanding certain concerns pertaining to the specificity of the TUNEL assay itself, it was demonstrated that the early stages of apoptosis, as detected by TUNEL, are capable of being reversed [22]. For instance, in 2001, Geske et al. [23] reported that the early stages of p53-induced apoptosis, as detected by TUNEL, in particular at 90 min, are reversible. TUNEL is therefore not a comprehensive and conclusive indicator of general cytotoxicity. To capture a broader spectrum of cell viability outcomes, we employed the NRU and MTS assays, both of which assess overall cell damage regardless of the underlying death mechanism [24]. In addition to being more inclusive, these assays are well-established, less subjective, and more reproducible across laboratories, features particularly advantageous in the context of a multicenter study. For this reason, we selected NRU and MTS to evaluate the potential cytotoxic effects of vanillin on HAECs in a standardized and replicable manner across the participating laboratories.

Moreover, we addressed the main limitation mentioned by the authors: flavor dilution not in propylene glycol (PG) or glycerol (VG), and heating without using an electronic cigarette and a standardized exposure mechanism. We prepared e-liquids by dissolving the vanillin in PG and VG solvents and heating the e-liquid through an electronic cigarette vaped using a standardized vaping machine. We maintained the composition of trapping solution (Ethanol/PBS – 20:80%), as indicated by the corresponding author. Fetterman et al. [16] evaluated cytotoxicity by conducting an exposure for only 90 min, which we considered too short to capture potential toxic effects fully. Therefore, we extended the exposure duration to 24 h to provide a more comprehensive evaluation.

Our results from both NRU and MTS revealed a cytotoxic effect related to the ethanol present in the vehicle control rather than the presence of vanillin in the aqueous extract. It is recognized that ethanol has a cytotoxic effect on cells, especially at higher concentrations [25]. This is why we included a vehicle control for each concentration used in the assays, demonstrating that the apparent cytotoxicity of PG/VG and PG/VG with vanillin was mainly due to the presence of ethanol in the trapping solution. Beyond Fetterman’s work, there are no other data in the literature focusing on the cytotoxicity of vanillin in endothelial cells. Furthermore, a recent study by Goenka S [26]. found that vanillin can affect wound healing on human retinal pigment cells, but not cytotoxicity [15]. However, in this study, e-liquids were directly applied to the cells, which suggests that the main limitation of this study is that this type of exposure differs from the real-world scenario.

Another important property of vanillin is its antioxidant and anti-inflammatory activity [27, 28]. Vanillin is reported as a potent scavenger of ROS and an inhibitor of inducible nitric oxide synthase (iNOS) [29]. Assessing oxidative stress, Fetterman and colleagues did not observe a significant increase in ROS after treatment with vanillin. Likewise, our results indicated that vanillin has no pro-oxidant effect on aortic endothelial cells. These results suggest that vanillin, as a component of e-liquids, may not contribute to oxidative stress, which is often a concern with inhalation products.

The bioavailability of nitric oxide (NO) is a critical determinant of endothelial function and plays a central role in maintaining vascular homeostasis with its strong vasodilatory, anti-inflammatory, and antioxidant properties [30]. Decreased NO levels have been implicated in endothelial dysfunction, leading to prothrombotic, proinflammatory, and less compliant blood vessel walls [30, 31]. Our investigation into the bioavailability of NO in aortic endothelial cells yielded intriguing results, as we found no significant changes in NO levels in endothelial cells treated with PG/VG vanillin. This contrasts with the reported decrease in NO bioavailability by Fetterman and colleagues, which highlighted discrepancies with existing literature data. Vanillin is well known as a cardioprotective substance [32]. It has been found to stimulate dose-dependent relaxation of isometric tensions during coronary artery contractions induced by different muscle contraction-inducing agents [33]. Furthermore, oral administration of vanillin in mice revealed a cardioprotective effect, reducing cardiac protein oxidation and lipid peroxidation and improving cardiac morphology [34]. Heating vanillin contained in e-liquids could lead to the formation of substances that could impair the endothelial NO balance state, but our results show that acute exposure to PG/VG vanillin under close-to-realistic conditions resulted in no reduction in aortic endothelial cells.

Likewise, the original study, we evaluated the expression of IL-6 and ICAM-1 genes in aortic endothelial cells under different experimental conditions, specifically focusing on the potential impact of PG/VG and PG/VG vanillin treatments. The outcomes of this study did not reveal any statistically significant variations in IL-6 and ICAM-1 gene expressions when compared to the control, regardless of both experimental settings (Regular or Sub-ohm) or the concentrations used (10 mM, 1 mM, 0.1 mM, and 0.01 mM). The evaluation of IL-6 gene expression under the specified conditions demonstrated no significant differences between the control and treated groups. This observation is consistent across both the Regular and Subohm experimental setups. These findings stand in contrast to the original study by Fetterman et al. (2018) [16], which reported an overexpression of IL-6 in aortic endothelial cells exposed to 10 mM vanillin. The lack of a significant effect in our replication study suggests that the method of flavor exposure could significantly influence the evaluation of IL-6 expression. The observed discrepancies could be supported by the results of other studies reporting anti-inflammatory properties of vanillin [27, 32, 35, 36].

Similar to IL-6, the gene expression of ICAM-1 did not show statistically significant variations under any treatment conditions compared to the control. This result was consistent across all concentrations and both experimental settings. The findings align with the original study by Fetterman et al. (2018) [16], which also did not observe any significant effects of vanillin on ICAM-1 gene expression. This consistency reinforces the conclusion that vanillin, at the tested concentrations, does not influence ICAM-1 expression in aortic endothelial cells.

Finally, while commonly used in various scientific applications, ethanol poses potential challenges that require careful consideration, especially when conducting in vitro experiments. Ethanol is an alcohol with two carbon atoms, characterized by good solubility in water and moderate polarity, which give it the ability to cross cell membranes easily. This property, together with its amphipathic nature, makes it a widely used solvent for solubilizing hydrophobic compounds or preparing extracts from complex matrices [37]. However, the same ability to interact with membranes and macromolecular structures [38] can contribute to its cytotoxic [25, 37, 39, 40] and pro-oxidant effects [20, 41], as well as potential interference with analytical systems, which are important aspects to consider when evaluating results. Ethanol has been shown to induce dose-dependent cytotoxicity in human umbilical vein endothelial cells (HUVECs) through mechanisms involving increased reactive oxygen species and depletion of antioxidant systems [20]. Similarly, Ayazoglu Demir et al. found that ethanol exerted dose-dependent cytotoxic effects in a variety of human cell lines, with different results depending on the cell type. Notably, this study stressed the importance of experimentally determining the solvent’s toxicity threshold for each cell type to avoid false-positive or false-negative results in cell-based assays [40]. In addition to its biological effects, ethanol can also compromise the repeatability and reproducibility of experimental results due to its volatility and concentration-dependent activity. Factors such as temperature and humidity and the presence of other solvent can influence evaporation rates [42, 43], leading to variations in sample concentrations and, consequently, inconsistent results. These factors, together with the intrinsic variability associated with a multicenter study design, may explain the poor reproducibility obtained in some of our experiments. While our results highlight the cytotoxic role of ethanol, further studies would be necessary to determine whether a similar effect applies to other flavoring agents.

Although this study was designed to conceptually replicate the work conducted by Fetterman et al. and address some of the main critical issues that emerged in the original work, several limitations should be acknowledged. First, as with all in vitro models, our experimental system does not fully replicate the complexity of human exposure. Despite the utilization of physiologically relevant endothelial cells and a standardized vaping system for aerosol generation, the in vitro environment is devoid of the biological integration present in vivo. This integration encompasses systemic metabolism and interactions between different cell types and tissues. Moreover, despite the attention paid to controlling vehicle effects and standardizing the exposure protocol, the use of ethanol as a collection solvent introduces potential variability related to its volatility and dose-dependent cytotoxicity. Although appropriate vehicle controls were included, variations in ethanol concentration during the experiment may have affected the reproducibility of some assays. In addition, the multicenter design of the study introduces further variability. Despite harmonized SOPs, operator-dependent differences (e.g. handling and pipetting accuracy) and specific laboratory conditions (e.g. temperature and humidity) may have influenced fluorescence-based and gene expression assays more markedly than viability measurements, contributing to the limited reproducibility observed for these endpoints. Furthermore, our study focused exclusively on acute exposure and therefore does not allow conclusions to be drawn about the cumulative or long-term effects of vanillin exposure. Also, the analysis focused on a single aromatic compound. While this allowed its specific effects to be isolated, it does not reflect the complexity of the chemical mixtures present in commercially available e-cigarette liquids. These limitations must be taken into account when interpreting the translational relevance of our findings.

Another limitation that deserves further clarification is the fact that two parameters, coil resistance (Ohm value) and PG/VG ratio, were varied simultaneously when comparing regular and sub-ohm settings. This design choice might limit mechanistic interpretation regarding the specific contribution of each parameter. However, our intent was not to isolate these variables per se, but rather to simulate two distinct and widely adopted vaping modalities: regular (MTL) and sub-ohm (DTL) vaping. These configurations represent real-world usage scenarios, where hardware settings and e-liquid formulations co-evolve to meet user preferences and device capabilities.

Therefore, the inclusion of both settings was necessary to capture the toxicological impact of different vaping styles as a whole, rather than deconstructing individual factors. While this limits internal comparison between the two settings, it enhances translational validity of the study. We acknowledge this limitation and encourage follow-up studies with factorial designs to dissect the specific roles of coil temperature and PG/VG ratios. Nonetheless, we believe our current approach provides a relevant and informative depiction of the toxicological variability across typical consumer vaping profiles. Finally, while cytotoxicity is a validated and reproducible endpoint commonly used in toxicological screening, we acknowledge that it may not capture more subtle or early-stage cellular alterations. In our multicenter setting, cytotoxicity (NRU and MTS) showed the highest inter-laboratory agreement, supporting its role as a reliable benchmark. However, the poor reproducibility observed for other endpoints such as oxidative stress and gene expression suggests that more sensitive or pathway-specific markers might be needed to detect less overt effects. Future studies could benefit from incorporating omics-based approaches or functional endothelial assays in controlled single-laboratory designs to enhance mechanistic insights and reduce technical variability.

In conclusion, our conceptual replication of the experiments conducted by Fetterman et al. [16], which focused on the impact of vanillin in e-liquids on endothelial cell function, revealed some disparities in the results compared to the original study. Despite our meticulous efforts to align with the specified experimental procedures, methodological discrepancies - particularly the use of a standardized exposure method closer to real vaping conditions and the choice to test the vehicle control at different concentrations - emerged as critical factors contributing to the observed variations. Our findings offer preliminary insight into the cardiovascular effects of vanillin exposure in an in vitro endothelial model simulating e-cigarette use. Although limited to a single compound, the results support the need for further investigations to refine our understanding of the potential health effects associated with flavorings in e-liquids. Careful consideration of methodological aspects, particularly the choice of assays and exposure methods, is critical for reliable interpretation of results in studies of this nature.

## Supplementary Information

Below is the link to the electronic supplementary material.

Supplementary Material 1

## Data Availability

The datasets supporting the conclusions of this article are available in the Zenodo repository, [10.5281/zenodo.13628515] (10.5281/zenodo.13628515).
