# Supplementary material for: Toxicological Evaluation of Vanillin Flavor in E-Liquid Aerosols on Endothelial Cell Function: Findings from the Replica Project
Source: Cardiovasc Toxicol. 2026 Apr 13;26(4):42. doi: 10.1007/s12012-026-10111-0 (PMC13070984; doi:10.1007/s12012-026-10111-0)
Supplement: Supplementary file 1 — Supplementary Material 1 [file 12012_2026_10111_MOESM1_ESM.docx]

**Supplementary Material**

**Toxicological evaluation of Vanillin Flavor in E-Liquid Aerosols on Endothelial Cell Function: Findings from the Replica Project**

R. Emma^1,2,§^, A. Sun^3,4§^, K. Partsinevelos^5^, S. Rust^6^, V. Volarevic^7,8,9^, R. Lesmana^10,11,12^, A. Giordano^4,13^, H. Goenawan^10,11,12^, M. I. Barliana^12,14^, A. Arsenijevic^7,8^, N. Kastratovic^7,8^, V. Markovic^7,9^, B. Spasic^8^, A. Distefano^5^, L. Orlando^5^, G. Carota^5^, R. Polosa^1,2^, M. Caruso^2,3,5,*^, G. Li Volti^2,5^.

1. Department of Clinical and Experimental Medicine, University of Catania, Via S. Sofia, 97, 95123, Catania (Italy)
2. Center of Excellence for the Acceleration of Harm Reduction (CoEHAR), University of Catania, Via S. Sofia, 97, 95123, Catania (Italy)
3. Department of Biology, College of Science and Technology, Temple University, Philadelphia, PA 19122, USA.
4. Sbarro Institute for Cancer Research and Molecular Medicine, Center for Biotechnology, College of Science and Technology, Temple University, Philadelphia, PA 19122, USA.
5. Department of Biomedical and Biotechnological Sciences, University of Catania, Via S. Sofia, 97, 95123 Catania (Italy);
6. ECLAT Srl, spin off of the University of Catania, Via. S Sofia 89, 95123 Catania (Italy);
7. Center for harm reduction of biological and chemical hazards, Faculty of Medical Sciences University of Kragujevac, 69 Svetozara Markovica 69 34000 Kragujevac, Serbia.
8. Department of Genetics, Faculty of Medical Sciences, University of Kragujevac, 69 Svetozar Markovic Street, Kragujevac, Serbia.
9. Department of Microbiology and Immunology, Faculty of Medical Sciences, University of Kragujevac, 69 Svetozar Markovic Street, Kragujevac, Serbia;
10. Department of Biomedical Sciences, Faculty of Medicine, Universitas Padjadjaran, Bandung, Indonesia.
11. Division of Biological Activity, Central Laboratory, Universitas Padjadjaran, Bandung, Indonesia.
12. Center of Excellence for Pharmaceutical Care Innovation, Universitas Padjadjaran, Bandung, Indonesia
13. Department of Medical Biotechnologies, University of Siena, Siena, Italy.
14. Department of Biological Pharmacy, Faculty of Pharmacy, Universitas Padjadjaran, Jl. Ir. Soekarno Km 21, Jatinangor, 45363, Indonesia.

**^§^** These authors contributed equally to this work

***Corresponding author**

Massimo Caruso

Department of Biomedical and Biotechnological Sciences University of Catania

Via S. Sofia, 97, 95123 Catania, Italy

[mascaru@unict.it](mailto:mascaru@unict.it)


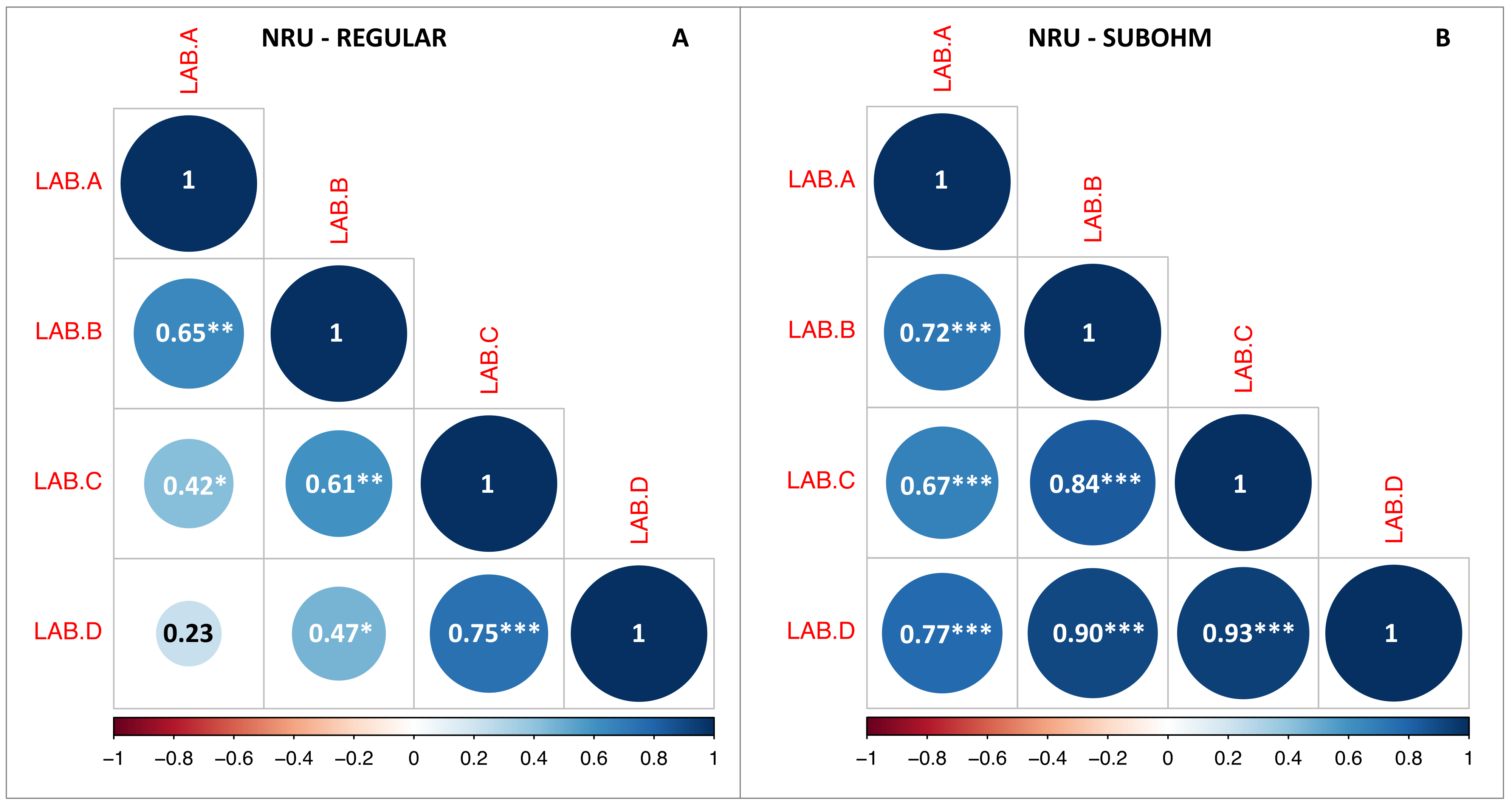


**Fig. S1** Correlograms of NRU data obtained from each laboratory. Panel (**A**) show correlation matrices for Regular setting; Panel (**B**) show correlation matrices for sub-ohm setting Each correlogram shows Spearman’s Rank correlation coefficients for all pairs of laboratory data as circles with the corresponding rho value. The color legend on the low side of the correlogram shows the correlation coefficients and the corresponding colors. Positive correlations are displayed in blue and negative correlations in red. The color intensity and the circle size are proportional to the correlation coefficient. Significant correlations were reported as follow: * p< 0.05; ** p< 0.01; *** p< 0.001


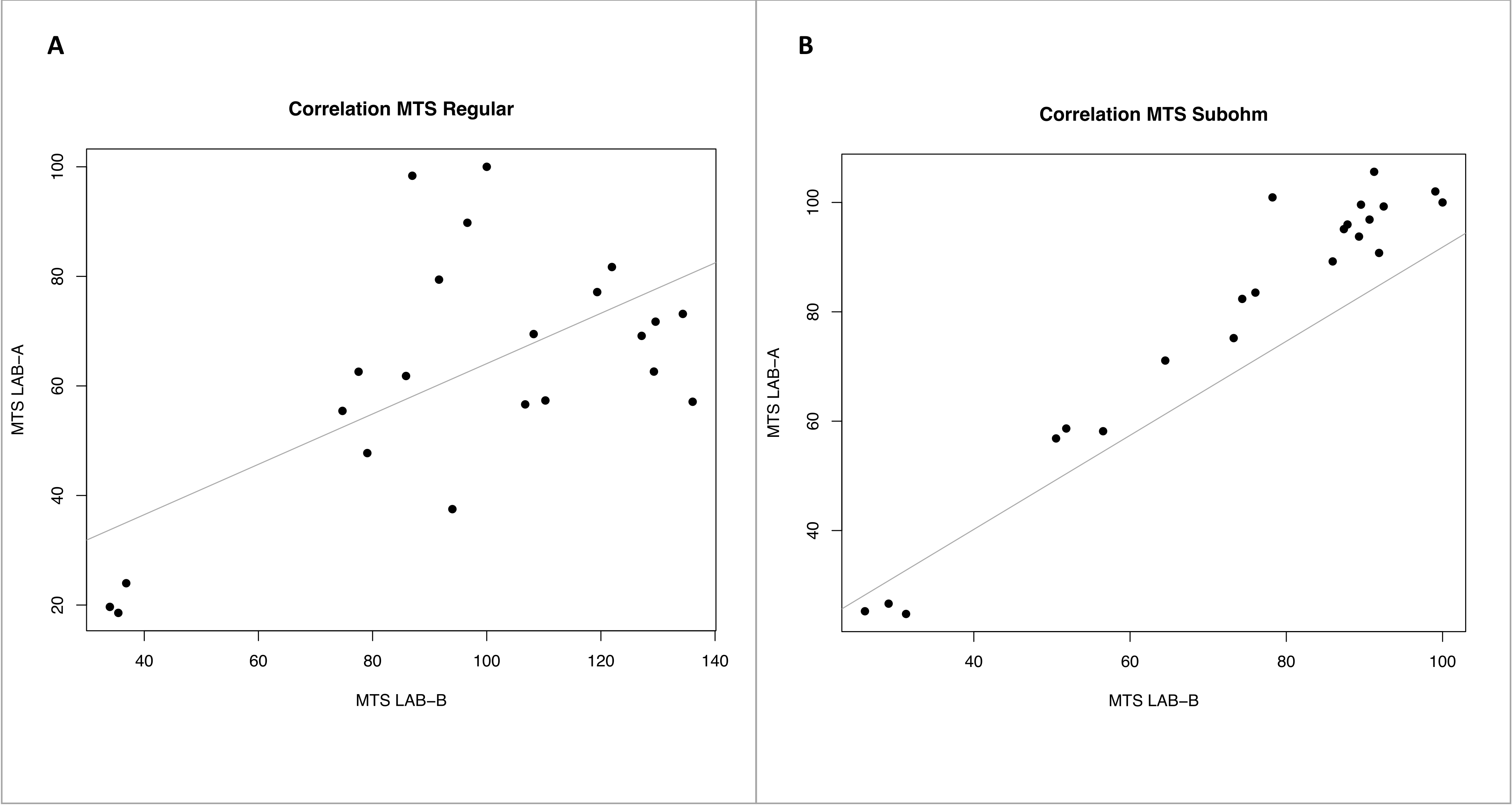


**Fig. S2** Correlation plots of MTS data for regular (**A**) and sub-ohm (**B**) settings. Spearman’s Rank correlation coefficients were 0.483 (p= 0.024) for regular setting and 0.896 (p< 0.001) for sub-ohm setting


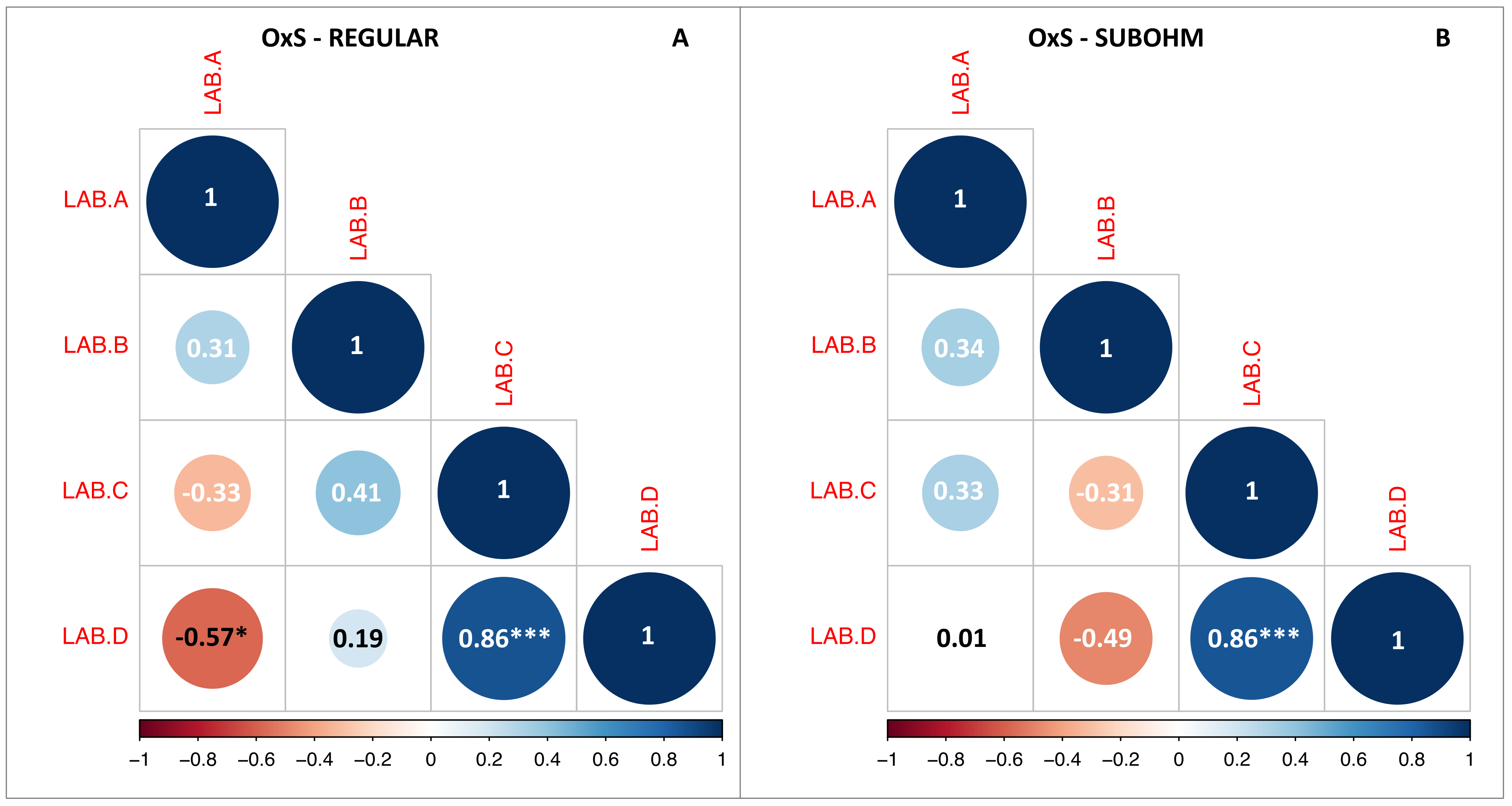


**Fig. S3** Correlograms of oxidative stress (OxS) data obtained from each laboratory. Panel (**A**) show correlation matrices for Regular setting; Panel (**B**) show correlation matrices for sub-ohm setting. Each correlogram shows Pearson’s correlation coefficients for all pairs of laboratory data as circles with the corresponding R value. The color legend on the low side of the correlogram shows the correlation coefficients and the corresponding colors. Positive correlations are displayed in blue and negative correlations in red. The color intensity and the circle size are proportional to the correlation coefficient. Significant correlations were reported as follow: * p< 0.05; ** p< 0.01; *** p< 0.001


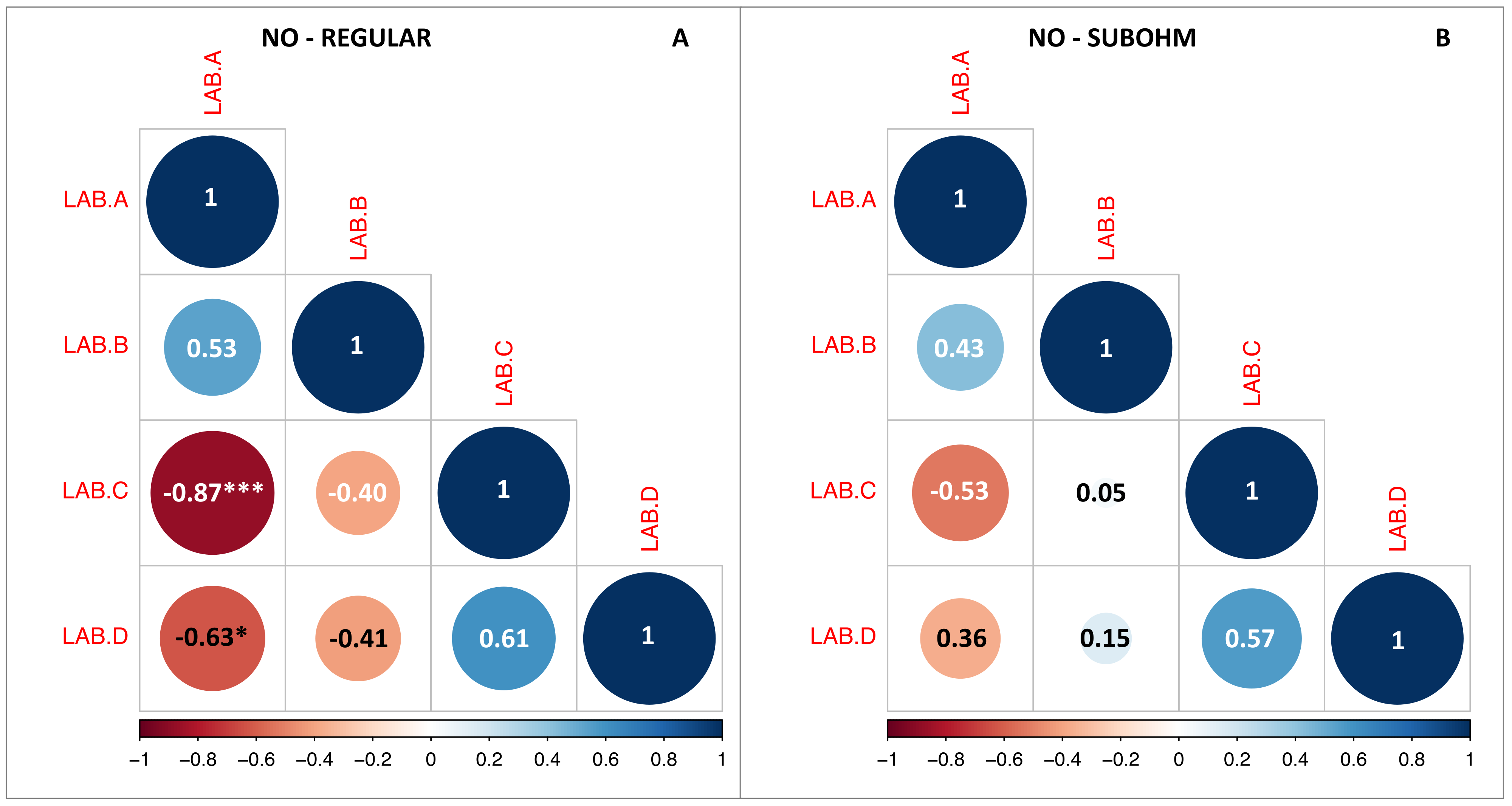


**Fig. S4** Correlograms of NRU data settings obtained from each laboratory. Panel (**A**) show correlation matrices for Regular setting; Panel (**B**) show correlation matrices for sub-ohm setting. Each correlogram shows Pearson’s (sub-ohm) and Spearman’s Rank (regular) correlation coefficients for all pairs of laboratory data as circles with the corresponding rho/R value. The color legend on the low side of the correlogram shows the correlation coefficients and the corresponding colors. Positive correlations are displayed in blue and negative correlations in red. The color intensity and the circle size are proportional to the correlation coefficient. Significant correlations were reported as follow: * p< 0.05; ** p< 0.01; *** p< 0.001.


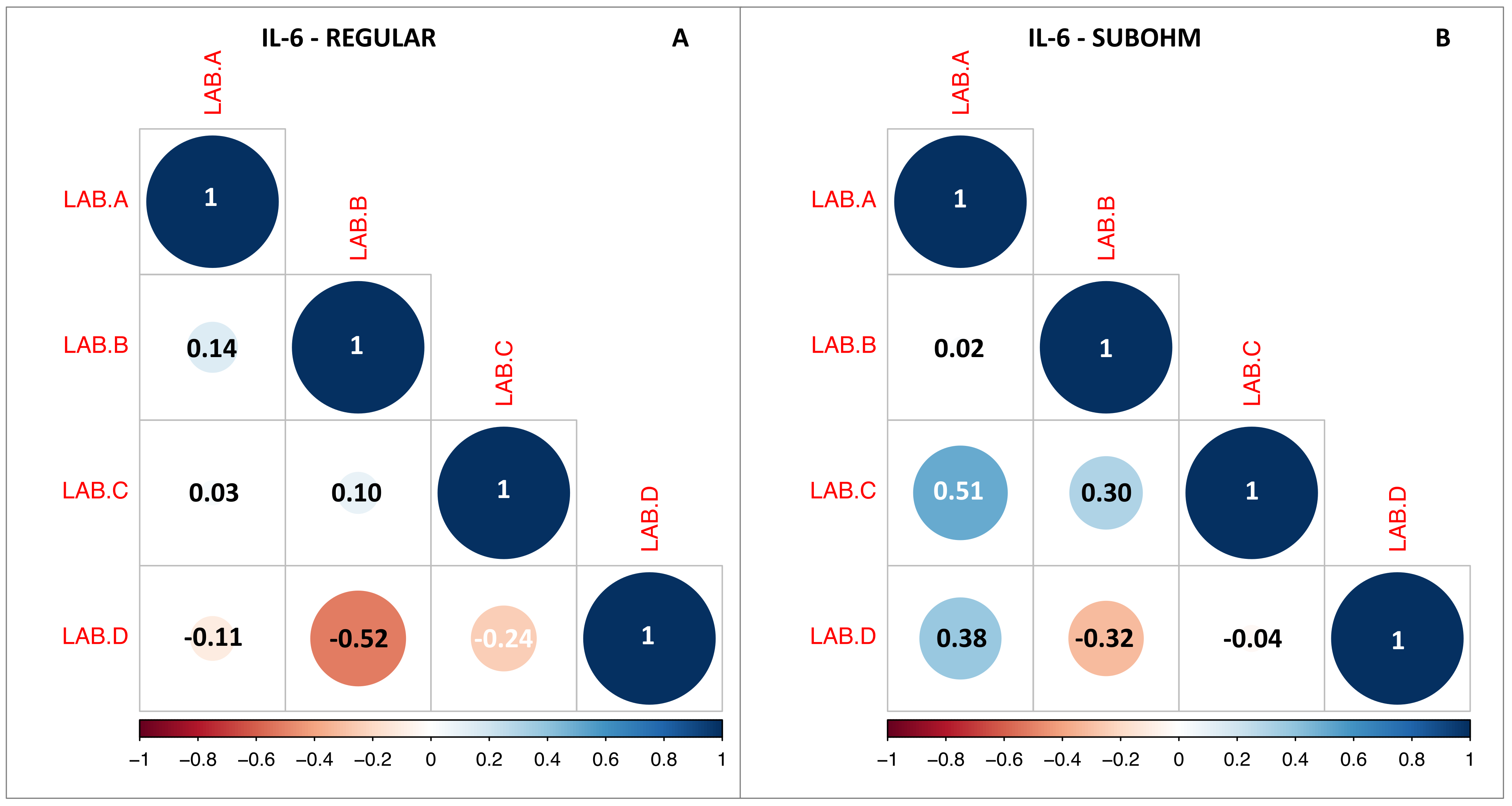


**Fig. S5** Correlograms of IL-6 gene expression data obtained from each laboratory. Panel (**A**) show correlation matrices for Regular setting; panel (**B**) show correlation matrices for sub-ohm setting. Each correlogram shows Spearman’s Rank correlation coefficients for all pairs of laboratory data as circles with the corresponding rho value. The color legend on the low side of the correlogram shows the correlation coefficients and the corresponding colors. Positive correlations are displayed in blue and negative correlations in red. The color intensity and the circle size are proportional to the correlation coefficient. No significant correlations were observed.


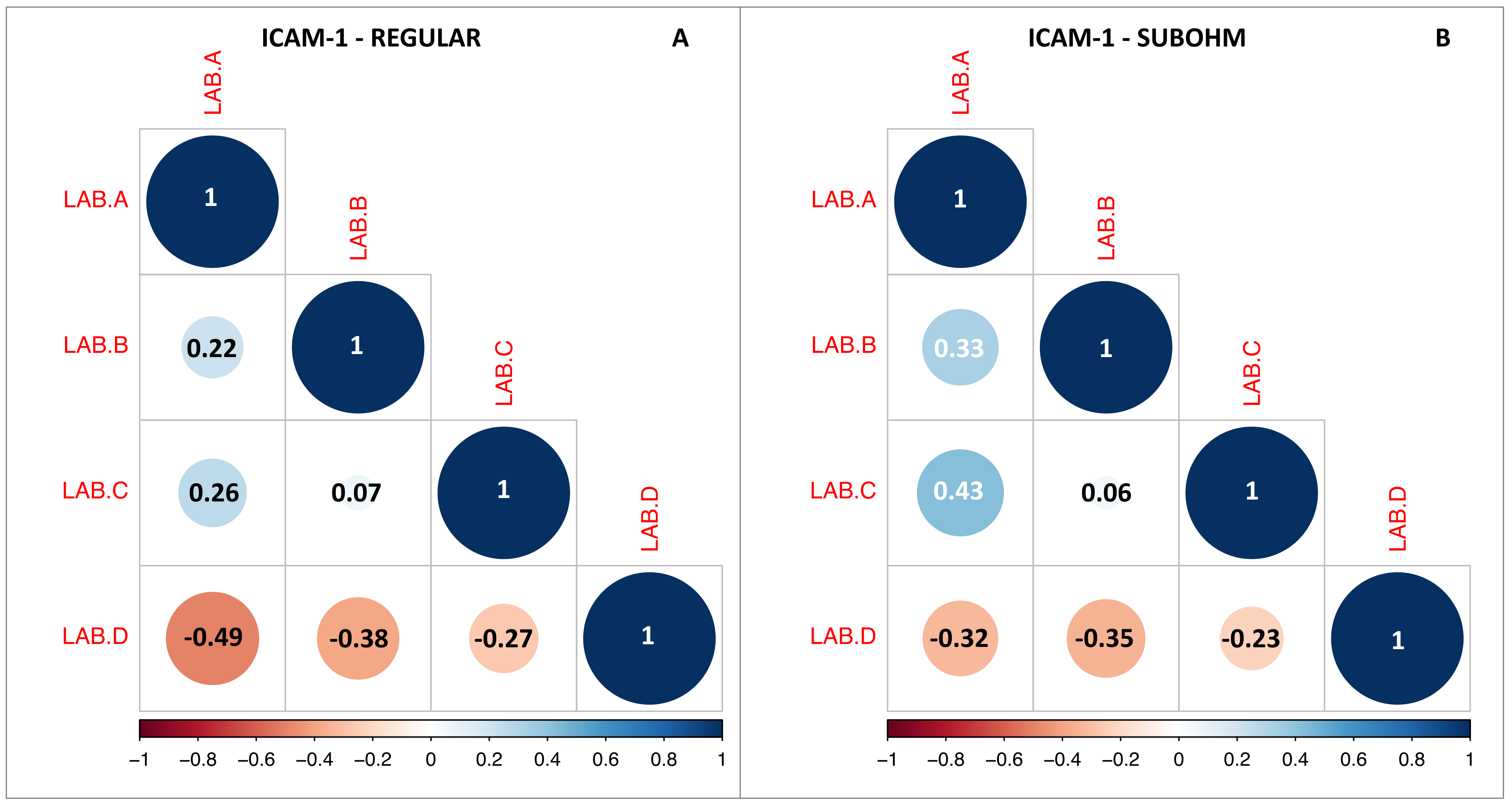


**Fig. S6** Correlograms of ICAM-1 gene expression data obtained from each laboratory. Panel (**A**) show correlation matrices for Regular setting; panel (**B**) show correlation matrices for sub-ohm setting. Each correlogram shows Pearson’s correlation coefficients for all pairs of laboratory data as circles with the corresponding R value. The color legend on the low side of the correlogram shows the correlation coefficients and the corresponding colors. Positive correlations are displayed in blue and negative correlations in red. The color intensity and the circle size are proportional to the correlation coefficient. No significant correlations were observed.

**Vanillin quantification in the aqueous extracts (AqEs)**

The vanillin concentration in the AqEs was determined using a colorimetric assay based on the reaction with o-toluidine [1]. A vanillin standard curve was prepared by serially diluting a 2 mg/ml stock solution in PBS/ethanol (80%/20%) to obtain concentrations ranging from 1 mg/ml to 15.625 µg/ml. Samples (500 µl) were then mixed with 100 µl of an o-toluidine/N,N-dimethylformamide solution (1:3, v/v), followed by the addition of 100 µl of Na2HPO4-citric acid buffer. The reaction mixture was then subjected to incubation at 100°C for a duration of 15 minutes, after which it was allowed to cool to room temperature. Absorbance was measured at 360 nm using a spectrophotometer, and vanillin concentration was interpolated from the standard curve.

The quantification of vanillin revealed detectable levels in AqEs generated using e-liquids containing vanillin flavoring. Conversely, no vanillin was detected (ND) in the AqEs generated using PG/VG e-liquids, irrespective of the device employed. In PG/VG Van samples, vanillin concentrations were 414.0 ± 73.8 µg/ml for the regular setting and 491.1 ± 49.5 µg/ml for the sub-Ohm setting (Table S1). These findings suggest that the sub-ohm device generates a higher vanillin yield compared to the regular device (p = 0.007), as showed in figure S7.

**Table S1.** Vanillin concentration in the aqueous extracts (AqEs)

|  | **Vanillin (ug/ml)** | |
| --- | --- | --- |
|  | **Regular** | **sub-Ohm** |
| **PG/VG** | ND | ND |
| **PG/VG Van** | 414.0 ± 73.8 | 491.1 ± 49.5 |

ND: not detectable

**Fig. S7** Vanillin concentration in the AqEs. Data are presented as the mean ± standard deviation (SD) of four independent experiments, each performed in triplicate. P value was calculated using T test.

**Evaluation of controls for the oxidative stress (OxS) assessment**

**Fig. S8** HAECs Oxidative Stress (OxS) evaluation by DHE of control medium, antimycin, and vehicle controls for the regular setting experiments. P values were calculated using pairwise Wilcoxon rank-sum test with Bonferroni correction. **p< 0.01 compared to control medium.

**Fig. S9** HAECs Oxidative Stress (OxS) evaluation by DHE of control medium, antimycin, and vehicle controls for the sub-ohm setting experiments. P values were calculated using pairwise Wilcoxon rank-sum test with Bonferroni correction. **p< 0.01, ***p< 0.001 compared to control medium.

**References**

[1] Zhao, J., Xia, H., Yu, T., Jin, L., Li, X., Zhang, Y., Shu, L., Zeng, L., & He, Z. (2018). A colorimetric assay for vanillin detection by determination of the luminescence of o-toluidine condensates. *PLoS One, 13*(4), e0194010. <https://doi.org/10.1371/journal.pone.0194010>
